# Understanding substrate substituent effects to improve catalytic efficiency in the SABRE hyperpolarisation process†
†Electronic supplementary information (ESI) available. See DOI: 10.1039/c9cy00396g


**DOI:** 10.1039/c9cy00396g

**Published:** 2019-07-10

**Authors:** Emma V. Stanbury, Peter M. Richardson, Simon B. Duckett

**Affiliations:** a Centre for Hyperpolarisation in Magnetic Resonance , Department of Chemistry , University of York , York , YO10 5NY UK . Email: simon.duckett@york.ac.uk

## Abstract

The use of *para*hydrogen based hyperpolarisation in NMR is becoming more widespread due to the rapidly expanding range of suitable target molecules and low-cost of *para*hydrogen production. Hyperpolarisation *via* SABRE catalysis employs a metal complex to transfer polarisation from *para*hydrogen into a substrate whilst they are bound. In this paper we present a quantitative study of substrate–iridium ligation effects by reference to the substrates 4-chloropyridine (**A**), 4-pyridinecarboxaldehyde methyl hemiacetal (**B**), 4-methylpyridine (**C**) and 4-methoxypyridine (**D**), and evaluate the role they play in the SABRE catalysis. Substrates whose substituents enable stronger associations yield slower substrate dissociation rates (*k*_d_). A series of variable temperature studies link these exchange rates to optimal SABRE performance and reveal the critical impact of NMR relaxation times (*T*_1_). Longer catalyst residence times are shown to result in shorter substrate *T*_1_ values in solution as binding to iridium promotes relaxation thereby not only reducing SABRE efficiency but decreasing the overall level of achieved hyperpolarisation. Based on these data, a route to achieve more optimal SABRE performance is defined.

## Abstract 2

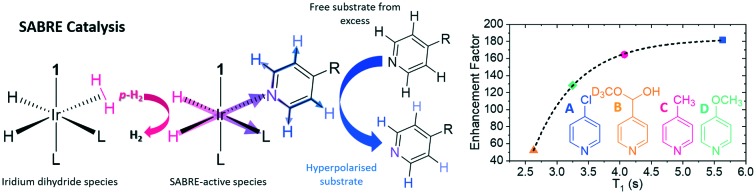
A quantitative study of substrate–iridium ligation effects identifies a route to achieve more optimal SABRE performance.

## Introduction

Nuclear magnetic resonance (NMR) is a powerful spectroscopic technique that provides detailed molecular and dynamic information. It has classically been used for the structural elucidation of complex natural products, proteins and macromolecules.^1^,^2^ However, NMR is inherently insensitive as the detected signal arises from the Boltzmann population difference across the nuclear spin states it probes.^3^ Practically, this means that only 1 in every 31 000 protons in a molecule contribute positively to the detected response in a 400 MHz spectrometer at 298 K.^4^ Although this population difference can be exacerbated by lowering the temperature,^5^ or using a larger magnetic field,^6^ such changes only induce relatively limited increases in the observed signal.

**Fig. 1:**
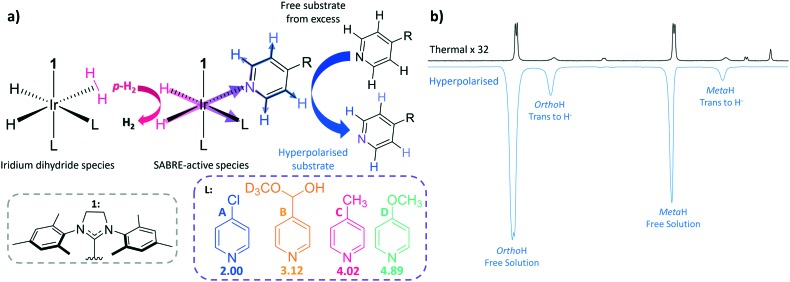
(a) Schematic of the SABRE process which brings protons that were previously located in *p*-H_2_ into spin–spin contact with the substrate (sub). Upon dissociation the NMR signals for the substrate become catalytically enhanced beyond their normal Boltzmann determined intensity levels. The SABRE-active species is [Ir(H)_2_(**1**)(L)_3_]Cl where substrates L, **A–D**, are differentiated according to R which is Cl, Me, OMe and CH(OH)(OCD_3_) respectively. (b) Typical ^1^H NMR spectrum resulting from hyperpolarisation (bottom) with thermal reference spectrum (top), recorded at 9.4 T for a 50 mM loading of 4-methylpyridine (**C**) with 5 mM of the [IrCl(COD)(**1**)] pre-catalyst in methanol-d_4_.

NMR sensitivity can be greatly increased *via* hyperpolarisation, which manipulates the spin state populations of molecules prior to detection such that they deviate from the Boltzmann distribution associated with the measurement field.^7^ There are a number of different methods which fall under this umbrella, but the three most common examples are; spin exchange optical pumping (SEOP),^8^–^10^ dynamic nuclear polarisation (DNP),^11^–^13^ and *para*hydrogen induced polarisation (PHIP).^14^–^16^ SEOP of noble gases has given the required signal strength to diagnose lung pathologies,^17^ whilst DNP of biomolecules has led to the observation of metabolism in tumours.^18^–^20^ In fact, DNP is capable of providing polarisation levels of up to 91% for ^1^H nuclei in 150 seconds and 70% ^13^C in 20 minutes.^21^ PHIP techniques originally involved the catalytic addition of *para*hydrogen (*p*-H_2_) into an unsaturated centre, typically an alkene or alkyne.^22^–^25^ A limitation of this approach is therefore reflected in the requirement for the dehydro-variant of the biomolecule of interest, although polarisation transfer into cleavable molecular tags is now being employed to circumvent this problem.^26^–^28^ Alternatively, signal amplification by reversible exchange (SABRE),^29^,^30^ is a PHIP^31^ technique that does not induce chemical change into the target molecule. SABRE is observed when a target molecule (analyte/substrate) and *p*-H_2_ are brought together by the temporary formation of a scalar coupled spin network,^32^–^35^ facilitated *via* an inorganic, iridium catalyst. If the *p*-H_2_ and the substrate molecule are held within the same plane, the non-equilibrium spin order of *p*-H_2_ can be readily transferred into the NMR-active nuclei of the substrate^36^*via* the maximised *trans* couplings at low magnetic field (Fig. 1) due to magnetic inequivalence.^37^ In fact, it can achieve signal enhancements of up to 63% ^1^H polarisation in just a few seconds.^29^,^38^ It is comparatively simple and inexpensive to produce *p*-H_2_ and thus to achieve significant enhancements, making SABRE an attractive candidate for use in industrial and clinical settings.

In SABRE, hyperpolarisation is transferred from the *p*-H_2_ to the bound substrate at low magnetic field when the correct resonance condition is satisfied. These conditions are well understood and nuclei dependent, for instance, transfer into ^1^H typically proceeds at around 65 G,^36^,^39^,^40^ whereas much lower mG fields are required for transfer into ^13^C (ref. 41) and ^15^N.^42^ After this point, the now hyperpolarised substrate dissociates from the iridium centre into solution. As *p*-H_2_ addition is reversible, the source of polarisation in the metal complex can be refreshed *via* an iridium dihydrogen dihydride species^32^,^40^,^43^ before a new substrate molecule from solution can associate to the metal centre for subsequent polarisation transfer (Fig. 1). This spontaneous polarisation process continues in the presence of *p*-H_2_ in a catalytic manner until the sample has been transferred to high field (*i.e.* into the spectrometer) for detection. Hence substrates are required to weakly coordinate to the metal centre as it is their reversible binding which allows for polarisation build-up in solution. The extent of polarised substrate created in solution is therefore in part controlled by the strength of the ligation between iridium and the substrate. For this reason, the most commonly exploited SABRE substrates are N-heterocycles, although nitriles,^44^ phosphines^45^ and diazirines^46^ have also been hyperpolarised. Fortunately, heterocyclic rings play a vital role in drug motifs^47^–^49^ and are heavily prevalent in biological systems.^50^,^51^ The hope is that the hyperpolarisation of these compounds could pave the way for their use as magnetic resonance imaging (MRI) contrast agents for the early diagnosis and treatment of disease in an analogous way to DNP polarised pyruvate.^18^–^20^ Recent SABRE developments have extended the substrate range to include amines, carboxylic acids and alcohols through the SABRE-RELAY variation which relies on a second proton exchange step.^52^,^53^

It has proven to be a common feature of SABRE catalysts that they contain an N-heterocyclic carbene (NHC). Consequently, the influence the NHC identity plays on iridium–substrate binding for the substrate pyridine has been studied extensively.^32^,^54^,^55^ In the associated investigations of Weerdenburg *et al.*^55^ and Lloyd *et al.*,^32^ the effect of changing the functional group attached to the central imidazole ring was explored. Both reported that the larger these groups are, the faster the rate of pyridine dissociation. Optimum rates of dissociation and polarisation levels were found when mesityl rings were attached to the imidazole group type NHCs (IMes and SIMes). Recently, Rayner *et al.*^38^ expanded this study by systematically modifying the functionality at the *ortho*, *meta* and *para*-positions of the mesityl ring in conjunction with altering the substituents on the NHCs imidazolium backbone. It was established that modifying the *ortho* and *para* positions significantly affects the rate of dissociation and hence the iridium–substrate binding. Furthermore, modifying the functional group on the imidazole ring dramatically changes SABRE performance, for example substituting hydrogen for chlorine proved to considerably slow down the rate of dissociation and improve the level of SABRE enhancement.

In this paper, we explore the effect of iridium–substrate ligation on SABRE efficiency as a function of substrate substitution. In order to achieve this, a range of *para*-substituted pyridines are utilised which have conjugate acid p*K*_a_ values of between 2.00 and 4.89 (Fig. 1) as determined in methanol-d_4_ using a literature method (see ESI†).^56^*Para*-substituted pyridines were chosen in this study to minimise the steric impact of the functional group changes as *ortho*-substituted pyridines have previously been shown to exhibit dramatic steric effects,^57^ although in methanol solution 4-pyridinecarboxaldehyde exists as the corresponding methyl hemiacetal (B). The influence of the substituent on iridium–substrate binding was probed *via* exchange spectroscopy (EXSY)^58^ and spin–lattice relaxation (*T*_1_) measurements. It was anticipated that substrates with substituents that add electron density undergo less proton dissociation leading to high conjugate acid p*K*_a_ values and therefore form stronger iridium–substrate ligations, resulting in slow substrate dissociation rates (*k*_d_).^43^,^59^,^60^ Binding to iridium has also been found to promote relaxation (*T*_1_), and therefore we expected to find shorter *T*_1_ values in the case of strong binding.^61^,^62^ Conversely, substrates with low p*K*_a_ values are predicted to form weaker iridium–substrate bonds, leading to high *k*_d_ values and longer *T*_1_ values. We therefore use p*K*_a_ to order the ligands for comparison in this study.

## Experimental

### Sample preparation

The SABRE method of transferring latent polarisation from *p*-H_2_ into a molecule of interest requires an intermediary binding of the two entities which is achieved by a catalyst. The SABRE pre-catalysts used here have the general form [IrCl(COD)(NHC)] where the N-heterocyclic carbene (NHC) is employed primarily to control the stability of the SABRE complex, as the binding needs to occur on a timescale which allows sufficient polarisation transfer, without being bound so long as to allow subsequent depolarisation through relaxation. In this work three different NHCs are used, which are 1,3-bis(2,4,6-trimethyl-phenyl)imidazole-2-ylidene (**1**), 1,3-bis(4-*tert*-butyl-2,6-dimethylphenyl)imidazole-2-ylidine (**2**) and 1,3-bis(2,4,6-trimethylphenyl)-4,5-dichloroimidazol-2-ylidine (**3**). The structures of which can be found in Fig. 1 (for **1**) and 3 (for **2** and **3**). On addition of an appropriate substrate, one which will weakly bind to the catalyst to allow the reversible nature of SABRE to be exploited, the substrate will displace the chlorine to leave the catalyst in the general form [Ir(COD)(NHC)Sub]Cl. The solvent used here was methanol-d_4_ unless stated otherwise. Prior to the addition of H_2_ the sample was degassed using a three step freeze–pump–thaw method using a bath of dry ice and acetone in order to remove any oxygen from the solution. Upon addition of H_2_ to the sample the COD hydrogenates to form cyclooctane allowing two more substrate ligands to bind to the iridium centre which now has the general form [Ir(NHC)(Sub)_3_(H)_2_]Cl. This is the fully active SABRE catalyst where the H_2_ and substrate molecules are in reversible exchange, allowing fresh *p*-H_2_ to be added and the polarisation to be built-up in solution with time. Another crucial element to SABRE hyperpolarisation is the use of a polarisation transfer field (PTF). The PTF is a magnetic field which is required to satisfy the resonance condition which allows the latent polarisation of the *p*-H_2_ to be efficiently transferred to the molecule of interest, which for transfer to ^1^H is a field of around 60 G (6 mT). The PTF used here was either produced from a hand-held magnetic array based on a Halbach design using permanent magnets to give a magnetic field of 61 G for the manual shaking method or *via* the use of a solenoid for the automated flow approach. The magnetic field is chosen such that the NMR *J*-coupling between the hydrides is equal to the chemical shift difference between the *p*-H_2_ derived hydrides to the bound substrate resonance of interest. In each case the sample activation was monitored by hyperpolarising the sample with 4 bar (absolute) *p*-H_2_ in a field of 61 G and observing the hydride resonances. This step was repeated until there was only a single hydride which corresponds to [Ir(NHC)(Sub)_3_(H)_2_]Cl as the hydrides are magnetically equivalent and thus produce a singlet in the resulting NMR spectra.

The p*K*_a_ values for the four substrates used in this study were determined by NMR titration according to a literature method (see ESI†).^56^

### Manual shaking method

In the manual shaking method NMR tubes fitted with Young's valves (GPE Scientific) were employed, such that the gas could be replaced between hyperpolarisation steps. Each time the sample was hyperpolarised the headspace of the NMR tube would be evacuated using a residual vacuum and subsequently refilled with fresh *p*-H_2_. In all cases the samples were shaken for 10 seconds using a 61 G magnetic field provided by a hand-held shaker,^63^ or the stray field of the NMR spectrometer, determined using a gauss meter. The hyperpolarisation spectra were acquired with a simple 90 degree pulse and acquire sequence with the addition of a suspend function at the beginning allowing the user to start the experiment the moment the sample is in the magnet. The speed of transfer here is on the order of 3 seconds between the end of sample shaking and the start of the sequence. The transfer time is important here as once the hyperpolarised signal has been established it will start to relax, therefore faster transfer times will yield larger signal enhancements. The source of *p*-H_2_ used in these experiments is a bespoke generator which is comprised of a cold head which uses a closed-circuit helium compression unit to reach temperatures as low as 7 K, however, at this temperature H_2_ is no longer a gas and therefore there is a feedback loop connected to a heater to maintain the temperature at 28 K. The H_2_ gas is then passed over a *para*-magnetic catalyst to allow conversion between the *para* and *ortho* hydrogen states. The purity of *p*-H_2_ is dependent on the interconversion temperature, at 28 K the purity of the resulting *p*-H_2_ is around 99%, which has been experimentally shown using this generator elsewhere.^64^

### Automated flow system

The drawback to using the aforementioned manual shaking method is that it is typically user dependent due to factors such as the vigour of the shaking and speed of the subsequent transfer into the spectrometer for detection. With this in mind, an automated flow system was designed which allows the control of all essential parameters, including the bubbling time, transfer time and magnetic field. This system allows for more reproducible data, especially when considering different users. However, typically this method provides much lower enhancements than the corresponding shaking method, this is attributed to the less vigorous mixing and the slower transfer time (on the order of 5 seconds). The flow system is comprised of a *p*-H_2_ generator (same generator used for the shaking method described above) set to 7 bar (absolute) pressure, an automated polarisation unit (Bruker) and a specially designed probe which will allow transfer of the solution into a high-field Bruker 400 MHz spectrometer after polarisation. The sample is housed inside a multi-layered glass cell which has been designed and built in-house, in between the layers there is a water/antifreeze mixture flowing around the chamber *via* a temperature controller (Huber Minichiller 300). The flow rate of this temperature controller is sufficiently fast, allowing accurate control of the temperature of the sample when it is outside the magnet in the range of 258–353 K. The flow samples contain the same proportions of materials as the corresponding manual shaking approach, however, they are scaled up from 0.6 mL to 3 mL as the volume of the chamber is larger than the NMR tube. Inside the mixing chamber is a porous frit connected to a glass tube which is supplied with *p*-H_2_ from the generator. The glass mixing chamber is positioned inside a solenoid, the magnetic field of which is controlled from the Bruker NMR software. To reduce the effect of solenoid heating, the field is only switched on for the duration of the bubbling of *p*-H_2_. All parameters are set within the NMR software giving the user complete control. This flow system has been previously described in detail for its use at both high-field and the adaptation to work with benchtop NMR spectrometers.^40^,^65^ More detail on the setup used here can be found in the ESI.†

### EXSY and *T*_1_ measurements

Ligation to the SABRE catalyst can be examined through the measurement of the dissociation rate of substrate binding to the catalyst. The method employed here to determine these so called *k*_d_ values is NMR exchange spectroscopy. This can be achieved by using selective shaped pulses applied to one of the bound resonances of the substrate and monitoring the evolution of the resulting signals over time. A set of 1D experiments were acquired, each with different delays to encode this behaviour. The integrals of the bound and corresponding free substrate peaks were recorded for these various delays. The chemical exchange model (presented in the ESI†) was used in conjunction with a least squares regression analysis to determine *k*_d_.

The longitudinal relaxation times (*T*_1_) of the substrate resonances have also been determined for these systems. This was carried out using a standard inversion recovery sequence or with the use of a hyperpolarised single-shot method. The rapid hyperpolarised single-shot method uses a train of variable flip angle pulses,^66^ that are separated by a delay to encode relaxation in a single hyperpolarisation step. By varying the flip angle in this way the same proportion of magnetisation is sampled each time and hence the resulting decay over time is caused by relaxation. Consequently, there is a trade-off between the amount of magnetisation that can be sampled per point and the number of points, in this case 15 points were acquired each with ∼25% signal when compared to a standard hyperpolarisation experiment (see ESI† for more detail).

## Results

### Enhancements

The four substrates 4-chloropyridine (**A**), 4-pyridinecarboxaldehyde methyl hemiacetal (**B**), 4-methylpyridine (**C**) and 4-methoxypyridine (**D**) shown in Fig. 1 were found to react with the precursor catalyst, [IrCl(COD)(**1**)] to form analogous SABRE active species. These were characterised by NMR spectroscopy (^1^H, ^13^C and ^15^N-NMR, see ESI†). Each yield a single hydride peak at ≊23 ppm which confirms the formation of octahedral products of the form [Ir(H)_2_(**1**)(sub)_3_]Cl, which contain chemically equivalent hydride ligands, that lie *trans* to two substrate ligands located in the equatorial plane. A third substrate ligand lies in the axial position, *trans* to the NHC (**1** in this case).

**Fig. 2:**
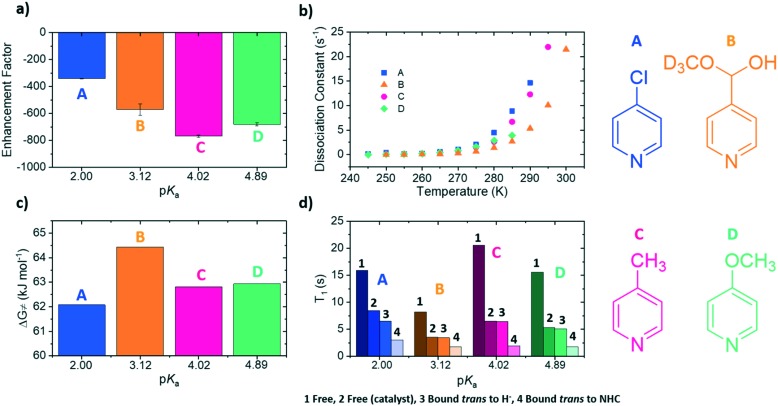
a) ^1^H NMR *ortho* proton SABRE enhancement achieved at 9.4 T for a 50 Mm concentration at 298 K [**A–D**], b) dissociation rate constant for ligand loss in the active SABRE catalyst as a function of temperature, c) Gibbs free energy of activation for ligand loss at 298 K and d) longitudinal ^1^H NMR relaxation time (*T*_1_) for the *ortho* proton site in each of the presented molecules measured at 9.4 T and 298 K. For each substrate in (d) there are four peaks labelled 1–4 which represent the *T*_1_ value for (1) the substrate free in solution without catalyst, (2) the substrate free in solution when the catalyst is present in solution, (3) the relaxation time when bound to the catalyst *trans* to the H^–^ and (4) the relaxation time when the substrate is bound to the catalyst *trans* to the NHC. The structures for each of the substrates labelled **A–D** have been included here to aid the reader.

In a typical SABRE experiment, a 5 mm NMR tube containing 5 mM of [IrCl(COD)(**1**)] and an excess of the target substrate (50 mM) is exposed to an atmosphere of *p*-H_2_ and shaken within a low magnetic field (≈65 G). This facilitates dissolution of *p*-H_2_ gas and the formation of the SABRE-active species; [Ir(H)_2_(**1**)(sub)_3_]Cl. Catalytic transfer of magnetisation then ensues from the *p*-H_2_ derived hydride ligands to the substrate which is then subsequently transferred into solution. All polarisation values were measured on a 400 MHz high field NMR spectrometer (Bruker Avance III). The four substrates **A–D** were examined in this way and exhibited ^1^H SABRE signal enhancements for all their proton resonances. Substrate **C** yielded the largest *ortho*^1^H NMR signal enhancement of –767-fold, compared to –340-fold (**A**), –680-fold (**D**) and –571-fold (**B**) (Fig. 2a).

### Rate of dissociation

If p*K*_a_ links to ligand binding, then there might be an optimum value. This is a reasonable hypothesis as in SABRE, the rate at which the substrate dissociates from the iridium centre contributes to the amount of polarised substrate in solution, and consequently the size of the signal enhancement. NMR exchange spectroscopy (EXSY) can be employed to probe this process.^58^,^59^ This involves a selective NOESY experiment in which the *ortho* protons on the substrate bound *trans* to the hydrides are selectively excited. This signal is monitored for a series of set mixing times to probe the amount of substrate that dissociates from the iridium centre. Dissociation rate constants (*k*_d_) were been determined for each of the four complexes over the temperature range 245–300 K (Fig. 2b, see ESI† for details) in this way. These rate data show that as the p*K*_a_ of the agent increases, the values of *k*_d_ vary inconsistently across the series which means this parameter is not a good indicator of binding potential.

**Fig. 3:**
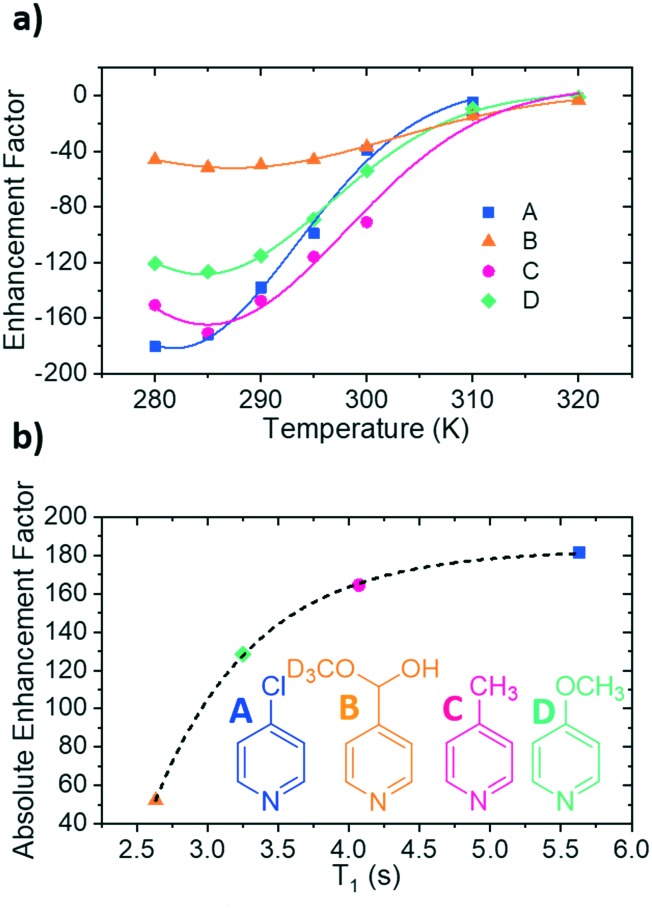
(a) Variation of ^1^H NMR signal enhancement for the *ortho* protons of the substrate as a function of temperature. (b) *T*_1_ values and corresponding magnitude of the enhancement factor at the optimum SABRE temperature for each substrate. The points have been fitted with a single exponential function, represented by the dotted line. The structures of the four substrates have been included as an inset of (b).

In 2016, Barskiy *et al.*^43^ suggested that the optimum dissociation rate for SABRE would be around 4.5 s^–1^. According to the Arrhenius equation, the rate of dissociation is temperature dependent and therefore the SABRE efficiency of these agents should also vary with temperature as their respective ligand dissociation rates approach this value. Using these variable temperature rate data allows the Gibbs free energy barrier to ligand dissociation (Δ*G*≠(298)) to be determined.^67^ The corresponding values of Δ*G*≠(298) are plotted in Fig. 2c as a function of the substrate conjugate base p*K*_a_ determined here in methanol. These data allow precise rates of ligand loss to be determined for a given temperature. The effect of temperature on the level of SABRE enhancement was explicitly probed using a variable temperature flow system (see ESI†) which though yielding poorer raw signal gains than the shake and drop method, is far more reproducible.^63^ These results were measured in 5 K increments between 280 K and 300 K (Fig. 3a). These discrete data points were then empirically modelled using a Gauss curve to estimate the temperature where optimum SABRE catalysis is achieved. These temperatures were subsequently used to estimate the rate of ligand loss at this point.

For **A**, an enhancement maximum was predicted for 282 K where the rate is 4.86 ± 0.79 s^–1^. The corresponding maxima were seen at 286 K for **C**, 285 K for **D** and 287 K for **B** where the analogous ligand exchange rates are 6.23 ± 1.01 s^–1^ (**C**), 5.15 ± 0.25 s^–1^ (**D**) and 3.96 ± 0.25 s^–1^ (**B**). These data confirm that stronger iridium–substrate associations generally suggest higher temperatures are required for optimum SABRE catalysis. However, whilst the associated optimal ligand loss rates are all comparable to the predicted 4.5 s^–1^ value of Barskiy, they are statistically different from one another.

### *T*_1_ relaxation

It is now understood that binding to the iridium centre promotes relaxation of the substrate protons and this additional effect might contribute to these small rate differences.^61^,^62^ This is reflected in the fact that the bound substrate exhibits shorter *T*_1_ values than the free material. The *T*_1_ values of the substrate protons in free **A–D** in solution were measured at 298 K for a 7-fold excess of free substrate, alongside the *T*_1_ values of the bound substrate; the corresponding values for these substrates were also measured without catalyst. These data are collected together in Fig. 2d.^62^,^68^

The protons that are located in the substrate *trans* to the NHC of the catalyst exhibit shorter *T*_1_ values than those of the related equatorial ligand. This is due to the strong iridium–nitrogen bond of the axial ligand, which consequently does not exchange on the timescale of these NMR. The *T*_1_ values for the equatorial substrate are clearly raised relative to their axial counterparts due to the contribution of the larger free material *T*_1_.^29^,^32^,^69^ These data also show that as the *k*_d_ increases, the *T*_1_ values decrease due to longer residence times on the catalyst. This reduction in *T*_1_ will contribute to the lower measured ^1^H NMR signal enhancement seen for **B** when compared to **C** and **D**.

The exchange weighted *T*_1_ values for each of the free substrates have also been measured in solution at the predicted optimum SABRE temperatures (Fig. 3b). For substrates **A–D**, these values are 5.63 ± 0.07 s, 2.6 ± 0.2 s, 4.07 ± 0.07 s and 3.3 ± 0.3 s respectively. These results confirm that once the rate of substrate dissociation is removed from consideration, *T*_1_ becomes the dominant factor controlling the level of SABRE enhancement observed. Fig. 3b shows explicitly that for longer *T*_1_ values larger enhancements can be achieved, and remarkably, these data points fit to a single exponential growth curve. This behaviour also highlights the benefit of longer *T*_1_ values on the resulting signal gain. The observed limit could be linked to variables such as the rate of hydrogen exchange/amount of *p*-H_2_ available.^32^

### Influence of the NHC

**Fig. 4:**
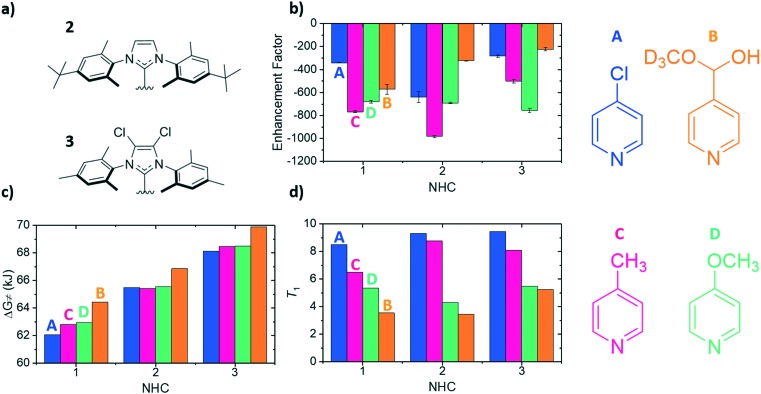
a) Structures of the carbene ligands 1,3-bis(4-*tert*-butyl-2,6-dimethylphenyl)imidazole-2-ylidine (**2**) and 1,3-bis(2,4,6-trimethylphenyl)-4,5-dichloroimidazol-2-ylidine (**3**), b) ^1^H NMR *ortho* proton SABRE enhancements using a substrate concentration of 50 mM [**A–D**], c) Gibbs free energy of activation values for ligand loss in the active SABRE catalyst at 298 K and d) corresponding longitudinal ^1^H NMR *ortho* proton relaxation times (*T*_1_) for the free substrate in the indicated substrate/catalyst combination. The chemical structures of each of the four substrates have been added for ease of understanding and in the cases of (b), (c) and (d) for each of the NHCs the corresponding values for each substrate are from right–left (**A–D**).

To compliment previous literature studies, measurements were expanded to include two additional NHCs to probe how the SABRE catalyst itself influences these data. The additional NHCs were 1,3-bis(4-*tert*-butyl-2,6-dimethylphenyl)imidazole-2-ylidine (**2**) and 1,3-bis(2,4,6-trimethylphenyl)-4,5-dichloroimidazol-2-ylidine (**3**) of Fig. 4a. Based on related literature,^38^ it was thought that NHC **2** would promote faster rates of substrate dissociation when compared to NHC **1** as it contains *tert*-butyl groups in the *para* position which were expected to add electron density to the iridium centre, thereby weakening the iridium–substrate associations. Conversely, NHC **3** should strengthen the iridium–substrate interaction as the chloride substituents produce a more electron-poor donor (supported by reported TEP values).^38^ In Fig. 4, the corresponding signal enhancements (a), Δ*G*≠(298) (b) and *T*_1_ values (c) are presented for each of the **A–D** substrates and catalysts containing NHC's **1–3**.

The largest overall signal enhancement was observed for the substrates **A** and **C** with NHC **2** (–981-fold and –640-fold respectively).^64^ However, substrate **B** worked best with NHC **1** (–401-fold signal enhancement) whilst **D** worked best with **3** (–756-fold) (Fig. 4a). Thus the NHC critically influences the iridium–substrate associations and subsequently, the associated enhancements. Enhancement values have been shown to increase if a deuterated form of the NHC is used.^62^,^68^ As substrate **C** achieved the largest enhancements with NHC **2**, its deuterated *d*_34_ analogue was assessed and found to result in a 30% increase in the enhancement factor (to –1411-fold, see ESI†).^64^

Fig. 4c demonstrates again that a substrate conjugate acids p*K*_a_ does not link to Δ*G*≠(298) as NHC **2** was predicted to encourage weaker iridium–substrate associations but these data actually confirm stronger associations exist. A similar trend is seen for NHC **3** which exhibits the highest Δ*G*≠(298) values of the series. Hence, steric contributions from the NHC to the substrate–iridium ligation must be considered when comparing these *para* substituted substrates and the different catalysts. The buried NHC ligand volumes of **1** (36.9%),^32^**2** (31.7%) and **3** (31.7%) quantify this difference and show that the catalyst form with NHC **1** is more sterically encumbered, meaning it promotes the formation of weaker iridium–substrate associations than those formed with **2** and **3**.

The *T*_1_ values for the free substrate were measured under these conditions (Fig. 4d). Their values followed the same trend seen previously for NHC **1**. For example, for NHC **1** the *T*_1_ values range from 8.49 s (**A**) to 3.54 s (**B**), for **2** they range from 5.46 s (**A**) to 4.31 s (**B**) and for **3** they range from 9.45 s (**A**) to 5.25 s (**B**). Despite the catalyst based on NHC **3** encouraging the formation of strong iridium–substrate ligations as demonstrated by the higher values of Δ*G*≠(298), the *T*_1_ values in this instance were higher than those with **1** and **2**. This could be a result of the change in complex rotational correlation time as a consequence of the heavier Cl atoms.

## Conclusions

SABRE catalysis is an important hyperpolarisation technique as it can produce significant signal enhancements cheaply and efficiently, without chemically changing the identity of the target molecule. In this paper we have explored the effects of substrate conjugate acid p*K*_a_ on SABRE to probe if this parameter can be simply linked to the efficiency of the SABRE process and find that even with these *para*-substituted materials no simple trend is followed. This prediction was based on the fact that one key parameter controlling SABRE is thought to be the residence time on the catalyst. If the substrate is bound on a short timescale then insufficient polarisation is built up, however for excessive ligation periods the polarisation will likely relax through normal NMR relaxation methods.

This study therefore involved using a range of structurally similar substrates to exemplify the effect on ligation to the catalyst. For example, substrate B (p*K*_a_ = 3.12) proved to bind stronger to the iridium centre in [Ir(H)_2_(**1**)(sub)_3_]Cl, as it exhibits slow dissociation (2.66 s^–1^) and a high Δ*G*≠(298) (64.44 kJ mol^–1^). Binding to iridium in this way promotes relaxation, therefore stronger associations result in shorter *T*_1_ values (3.54 s), since relaxation destroys hyperpolarisation, smaller signal enhancements were observed (–571 ± 41 fold). Contrastingly, substrate **A** (p*K*_a_ = 2.00) forms a weaker association (Δ*G*≠(298) = 62.08 kJ mol^–1^), and therefore dissociates on a faster timescale (8.88 s^–1^). This means that insufficient polarisation can be transferred prior to dissociation, resulting in smaller signal enhancements (–340 ± 2 fold). Large signal enhancements are observed where substrate–iridium ligation allows for sufficient polarisation to be transferred before relaxation effects are induced or substrate dissociation occurs, as in the case of substrate **C** (–767 ± 8 fold). In previous studies carried out elsewhere it has been suggested that an optimum rate of dissociation of around 4.5 s^–1^ exists.^43^ From the dissociation rates measured here *via* NMR as a function of temperature, it was observed that all substrates exhibited *k*_d_ values of around 4.5 s^–1^ at temperatures in the range 285–287 K. Subsequent measurements were taken using an automated flow system with temperature control to measure the SABRE enhancements as a function of temperature. It was observed that for each substrate the highest enhancement was achieved at temperatures coinciding with a *k*_d_ comparable to 4.5 s^–1^, thus agreeing with the previous works. The NMR relaxation time was measured at the optimal temperature for each substrate and compared to the respective enhancements at those temperatures. The result of this showed that increasing *T*_1_ yielded larger SABRE enhancement which suggests that once the ligation effects are optimised for any particular substrate, the *T*_1_ value becomes the dominant limiting factor. Interestingly, these data fit to a single exponential decay function, implying that the SABRE enhancement will not increase indefinitely with increasing *T*_1_. Beyond this point, other factors will limit the SABRE effect which we suggest may be linked to *p*-H_2_ concentration. The NHC ligand on the catalyst can also be used to control the dissociation rate. Here a further two catalysts (using NHC's **2** and **3**) were employed to highlight this effect. The largest signal enhancements are observed when Δ*G*≠(298) lies between 64.44 kJ mol^–1^ (sub **B**, NHC **1**) and 65.56 kJ mol^–1^ (sub **D**, NHC **2** – this range also includes **A** + **2** and **C** + **2**). However, for substrates **B** (NHC **1**) and **D** (NHC **2**) this binding results in short relaxation times (≈4 s). Contrastingly, substrates **A** and **C** exhibit longer relaxation times of 9.31 s and 8.76 s respectively. However, these values could be extended by deuteration of the NHC. This modification has also been seen to result in larger enhancements.^62^,^68^ For example, the deuterated analogue of NHC **2** induced a 30% increase in the enhancements for substrate **A** compared to the non-deuterated (see ESI†).^64^

This research confirms that by varying the SABRE catalyst in order to achieve the optimal dissociation rate of ∼4.5 s^–1^, it is possible to facilitate efficient hyperpolarisation transfer. Under these conditions however, relaxation acts to tension the final hyperpolarisation level thereby influencing the optimum rate. Furthermore, we find that *k*_d_ does not simply correlate with a substrates conjugate acids p*K*_a_ value and that steric effects must certainly be considered, even with these *para*-substituted agents, otherwise over interpretation is possible as it is the rate of ligand loss that is critical.

## Conflicts of interest

There are no conflicts to declare.

## Supplementary Material

Supplementary information
