# Supplementary material for: Understanding substrate substituent effects to improve catalytic efficiency in the SABRE hyperpolarisation process
Source: Catal Sci Technol. 2019 Jul 10;9(15):3914–22. doi: 10.1039/c9cy00396g (PMC6836623; doi:10.1039/c9cy00396g)
Supplement: Supplementary file 1 [file CY-009-C9CY00396G-s001.pdf]

Electronic Supplementary Material (ESI) for Catalysis Science & Technology.
This journal is © The Royal Society of Chemistry 2019

Supplementary Data

Understanding substrate substituent effects to improve catalytic efficiency in the SABRE hyperpolarisation process

Emma V. Stanbury, Peter M. Richardson and Simon B. Duckett

Contents

Supplementary Data 1
1. Characterisation of $[Ir(H)_2(NHC)(Sub)_3]Cl$ 2
A – 4-Chloropyridine 2
B – 4-Methylpyridine 3
C – 4-Methoxypyridine 3
D – 4-Pyridinecarboxaldehyde 3
2. Signal Enhancements 5
Calculation 6
Data 6
Predicting the enhancement for 4-methylpyridine (B) and catalyst 2 6
3. Substrate Dissociation 9
Exchange Pathways 9
Data 11
4. Temperature Enhancements 12
5. Calculating Gibbs free energy 14
6. T_1 relaxation 15
Calculation 15
Catalyst 1 15
7. pK_a Measurements Using NMR 18
8. References 24

1. Characterisation of [Ir(H)$_2$(NHC)(Sub)$_3$]Cl

In this work all the pre-catalysts used have the general form [IrCl(COD)(NHC)] where COD is 1,5 cyclooctadiene and the NHC is the stabilising N-heterocyclic carbene ligand. On addition of a substrate in a solvent the pre-catalyst then converts into a product of general form [Ir(COD)(NHC)(Sub)]Cl having displaced the chloride in favour of the substrate. On addition of H_2 the COD will then hydrogenate forming cyclooctane and be replaced on the catalyst with substrate molecules to form a dihydride complex of general form [Ir(H)$_2$(NHC)(Sub)$_3$]Cl (shown in Figure S1). This form of the catalyst will undergo reversible exchange of both H_2 and substrates in free solution thus allowing the SABRE process to work efficiently. The pre-catalyst is exposed to the *p*-H_2 gas via vigorous shaking for 10 seconds in a magnetic field of 61 G prior to being inserted into the magnetic for detection. This produces SABRE enhanced hydride resonances whose peaks in the hydride region correspond to hydrogen atoms in the aforementioned SABRE active catalyst [Ir(H)$_2$(NHC)(Sub)$_3$]Cl. The sample is considered fully activated once the hydride peaks corresponding to [Ir(H)$_2$(NHC)(Sub)$_3$]Cl no longer grow in intensity. Typically this takes around 15 minutes, but can vary slightly depending on the substrate used. These SABRE-active species, for substrates **A**-**D**, have been characterised for NHC **1** and are shown below. The substrate resonances, bound *trans* to hydride and *trans* to the NHC are listed alongside the hydride chemical shift as there are diagnostic of these products.[1]

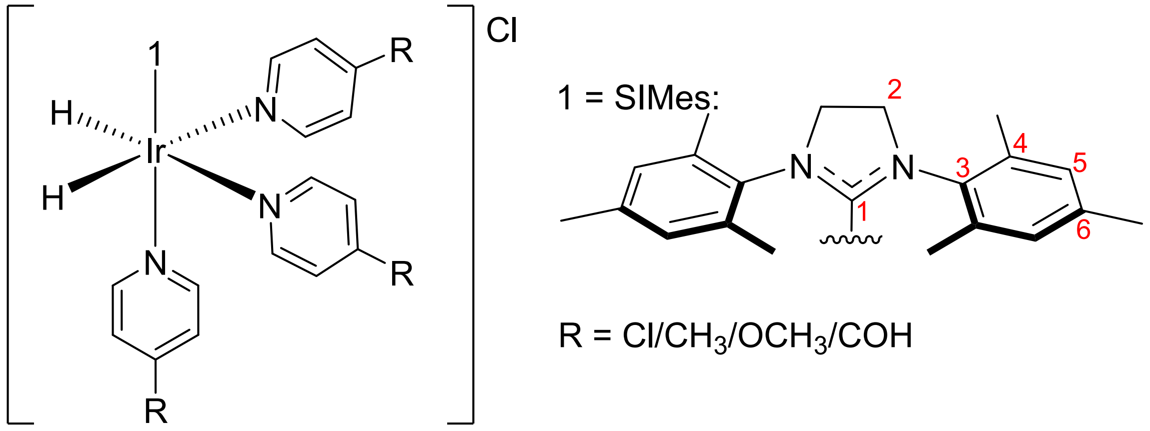

Figure S1 – General structure for [Ir(H)$_2$(NHC)(sub)$_3$]Cl

A – 4-Chloropyridine

^{1}H NMR (500 MHz, methanol-d$_4$, 235 K): δ 8.23 (d, 4H, *J* = 6.3 Hz, *ortho*H substrate *trans* to hydride), 8.00 (d, 2H *J* = 6.7 Hz, *ortho*H substrate *trans* to SIMes), 7.26 (d, 4H, *J* = 6.3 Hz, *meta*H substrate *trans* to hydride), 7.26 (d, 2H, *J* = 6.7 Hz, *meta*H substrate *trans* to SIMes), 6.64 (s, 4H, SIMes 5), 3.90 (m, 4H, SIMes 2), 2.25 (s, 12H, *ortho*CH_3^{Mes}), 2.18 (s, 6H, *para*CH_3^{Mes}), -22.53 (s, 2H, hydride).

^{13}C NMR (125 MHz, methanol-d$_4$, 235 K): δ 156.2 (*ortho*C substrate *trans* to SIMes), 155.1 (*ortho*C *trans* to hydride), 144.6 (*para*C substrate *trans* to SIMes), 144.0 (*para*C substrate *trans* to hydride), 138.0 (SIMes 4), 137.5 (SIMes 6), 135.9 (SIMes 3), 128.7 (SIMes 5), 125.9 (*meta*C substrate *trans* to SIMes), 125.8 (*meta*C *trans* to hydride), 50.3 (SIMes 2), 19.7 (*ortho*CH_3^{Mes}), 17.7 (*para*CH_3^{Mes}).

^{15}N NMR (41 MHz, methanol-d$_4$, 235 K): δ 250.6 (substrate *trans* to hydride), 236.3 (substrate *trans* to SIMes).

B – 4-Methylpyridine

^{1}H NMR (500 MHz, methanol-d_4, 235 K): δ 8.12 (d, 4H, *J* = 5.9 Hz, *ortho*H substrate *trans* to hydride), 7.80 (d, 2H *J* = 5.5 Hz, *ortho*H substrate *trans* to SIMes), 6.96 (d, 4H, *J* = 5.9 Hz, *meta*H substrate *trans* to hydride), 6.81 (d, 2H, *J* = 5.5 Hz, *meta*H substrate *trans* to SIMes), 6.61 (s, 4H, SIMes 5), 3.87 (m, 4H, SIMes 2), 2.24 (s, 12H, *ortho*CH_3^{Mes}), 2.34 (s, 3H, CH_3 substrate trans to SIMes), 2.17 (s, 6H, CH_3 substrate *trans* to hydride), 2.16 (s, 6H, *para*CH_3^{Mes}), -22.43 (s, 2H, hydride).

^{13}C NMR (125 MHz, methanol-d_4, 235 K): δ 154.2 (*ortho*C substrate *trans* to SIMes), 153.6 (*ortho*C *trans* to hydride), 147.9 (*para*C substrate *trans* to hydride), 148.6 (*para*C substrate *trans* to SIMes), 138.5 (SIMes 4), 137.1 (SIMes 6), 135.9 (SIMes 3), 128.7 (SIMes 5), 125.9 (*meta*C substrate *trans* to SIMes), 125.9 (*meta*C *trans* to hydride), 50.5 (SIMes 2), 19.8 (*ortho*CH_3^{Mes}), 19.5 (CH_3 substrate *trans* to SIMes), 19.4 (CH_3 substrate *trans* to hydride), 17.7 (*para*CH_3^{Mes}).

^{15}N NMR (41 MHz, methanol-d_4, 235 K): δ 247.2 (substrate *trans* to hydride), 233.1 (substrate *trans* to SIMes).

C – 4-Methoxypyridine

^{1}H NMR (500 MHz, methanol-d_4, 235 K): δ 8.08 (d, 4H, *J* = 6.5 Hz, *ortho*H substrate *trans* to hydride), 7.74 (d, 2H *J* = 6.9 Hz, *ortho*H substrate *trans* to SIMes), 6.69 (d, 4H, *J* = 6.5 Hz, *meta*H substrate *trans* to hydride), 6.56 (d, 2H, *J* = 6.9 Hz, *meta*H substrate *trans* to SIMes), 6.64 (s, 4H, SIMes 5), 3.87 (s, 6H, CH_3 substrate *trans* to hydride), 3.86 (m, 4H, SIMes 2), 3.72 (s, 3H, CH_3 substrate *trans* to SIMes), 2.25 (s, 12H, *ortho*CH_3^{Mes}), 2.17 (s, 6H, *para*CH_3^{Mes}), -23.01 (s, 2H, hydride).

^{13}C NMR (125 MHz, methanol-d_4, 235 K): δ 165.7 (*para*C substrate *trans* to hydride), 165.4 (*para*C substrate *trans* to SIMes), 155.7 (*ortho*C substrate *trans* to SIMes), 155.0 (*ortho*C *trans* to hydride), 138.6 (SIMes 4), 136.9 (SIMes 6), 136.0 (SIMes 3), 128.6 (SIMes 5), 110.9 (*meta*C substrate *trans* to SIMes), 110.8 (*meta*C *trans* to hydride), 50.8 (CH_3 substrate *trans* to hydride), 19.8 (*ortho*CH_3^{Mes}), 17.9 (*para*CH_3^{Mes}).

^{15}N NMR (41 MHz, methanol-d_4, 235 K): δ 232.9 (substrate *trans* to hydride), 218.0 (substrate *trans* to SIMes).

D – 4-Pyridinecarboxaldehyde

Note than upon addition of methanol-d_4, the methyl hemiacetal analogue of 4-pyridinecarboxaldehyde forms.

^{1}H NMR (500 MHz, methanol-d_4, 243 K): δ 8.27 (d, 4H, *J* = 5.1Hz, *ortho*H substrate *trans* to hydride), 7.97 (d, 2H *J* = 6.1 Hz, *ortho*H substrate *trans* to SIMes), 7.18 (d, 4H, *J* = 5.1 Hz, *meta*H substrate *trans* to hydride), 7.04 (d, 2H, *J* = 6.1 Hz, *meta*H substrate *trans* to SIMes), 6.60 (s, 4H, SIMes 5), 3.89 (m, 4H, SIMes 2), 2.24 (s, 12H, *ortho*CH_3^{Mes}), 2.16 (s, 6H, *para*CH_3^{Mes}), -22.39 (s, 2H, hydride).

^{13}C NMR (125 MHz, methanol-d_4, 243 K): δ 154.7 (*ortho*C substrate *trans* to SIMes), 154.0 (*ortho*C *trans* to hydride), 150.7 (*para*C substrate *trans* to SIMes), 150.0 (*para*C substrate *trans* to hydride), 139.7 (SIMes 4), 137.8 (SIMes 6), 135.8 (SIMes 3), 128.7 (SIMes 5), 122.6 (*meta*C substrate *trans* to SIMes), 122.5 (*meta*C *trans* to hydride), 50.3 (SIMes 2), 19.8 (*ortho*CH_3^{Mes}), 17.7 (*para*CH_3^{Mes}).

^{15}N NMR (41 MHz, methanol-d_4, 243 K): δ 254.2 (substrate *trans* to hydride), 240.1 (substrate *trans* to SIMes).

2. Signal Enhancements

Signal enhancements were measured using the both the shake and drop method and the automated flow approach to measure the enhancements at different temperatures (see main text).

Typically, samples were formulated by mixing 5 mM (1.92 mg) of catalyst and 50 mM of substrate in 0.6 mL of methanol-d_4. These were then degassed using a freeze-thaw method to ensure no air remained in the headspace of the sample. Hydrogen (4 bar) was then added to each sample, which was subsequently shaken to dissolve it in the solution, facilitating the formation of the SABRE-active species; $[Ir(H)_2(NHC)(sub)_3]Cl$. Once fully activated, any remaining gas was removed and *para*-hydrogen (99.9 %, 4 bar) was added. In order to achieve efficient SABRE polarisation transfer a polarisation transfer field (PTF) has to be used. For the shake and drop method the sample was either shaken in the stray field of the magnet (~65 G) or in a hand-held magnetic shaker (~61 G) for 10 seconds before being dropped into the spectrometer for FID acquisition. The stray field of the spectrometer was determined using a Gaussmeter. However, the stray field is inhomogeneous although the magnet is wide bore and has a reasonable volume over which a 65 G shake can be made. The hand-held shaker though provides a much better assurance of the polarisation field.[2] The hand-held shaker was therefore predominantly used in this research, however, when comparing the two methods the hand-held shaker gave only slightly better results, and this was attributed to the broad PTF dependence on the SABRE enhancement level for these substrates.

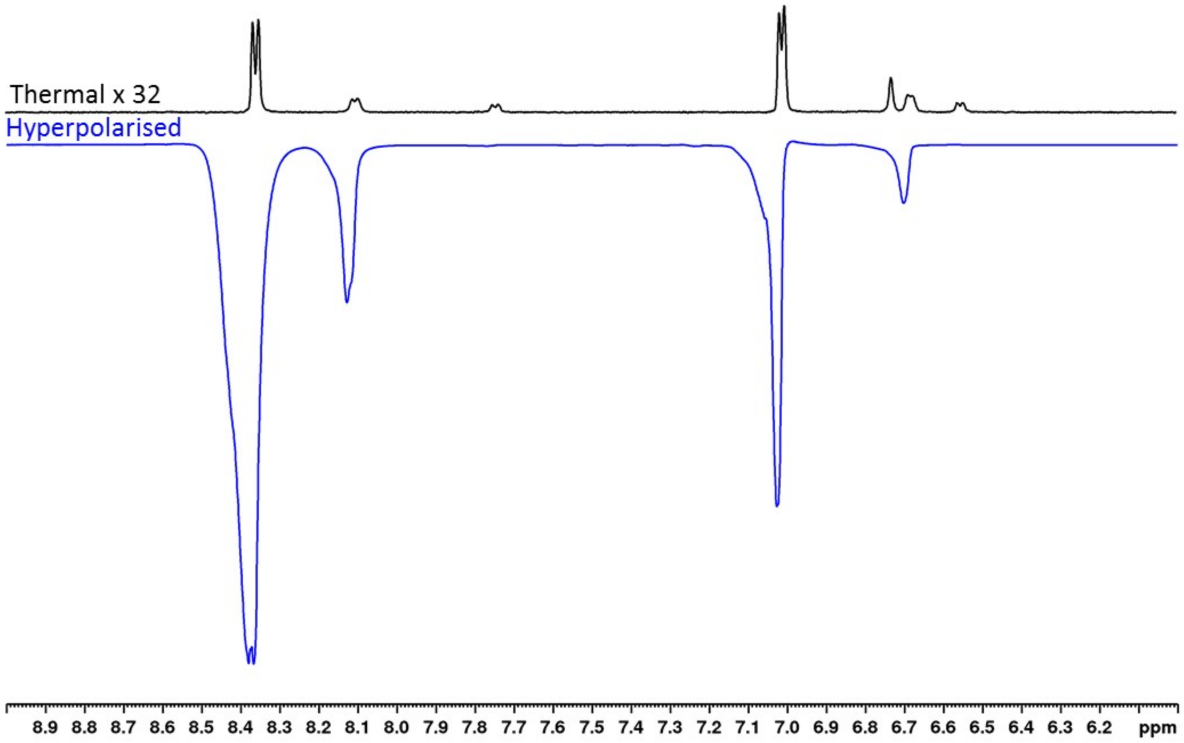

Figure S2 - Example of a hyperpolarised spectrum measured using the shake and drop method

Please note that for substrate **A**, 4-chloropyridine hydrochloride was used. Therefore, cesium carbonate (50 mM) was also added to this sample to remove the hydrochloride salt so that the nitrogen centre of the substrate was available to bind to iridium.

Calculation

The ^{1}H NMR signal enhancements were calculated using Equation S1.

$$\varepsilon_S = \frac{S_{hyp}}{S_{thermal}} \quad \text{(S1)}$$

Where ε_S, is the hyperpolarised enhancement factor of substrate, S_{hyp} is the integral of hyperpolarised sample and $S_{thermal}$ is the integral of thermal (reference) sample.

Data

The enhancements for the substrates A-D and catalysts 1-3 are shown below.

	Enhancement Factor		
Substrate	1	2	3
A	-340.31 ± 2.02	-637.60 ± 48.55	-282.33 ± 10.17
B	-767.04 ± 8.26	-980.18 ± 10.00	-499.50 ± 16.60
C	-680.20 ± 11.41	-690.57 ± 6.23	-755.71 ± 18.02
D	-571.41 ± 41.34	-323.32 ± 3.24	-222.96 ± 13.48

Table S1 - Enhancement factors for each of the substrate and catalyst combinations

Predicting the enhancement for 4-methylpyridine (B) and catalyst 2

It has been seen in previously investigated SABRE systems that very efficient polarisation transfer can cause radiation dampening effects resulting in anti-phase spectra.[3] When 4 bar of 99.9 % *para*-hydrogen was added to the complex $[Ir(H)_2(\mathbf{2})(\mathbf{B})_3]Cl$, radiation dampening effects were observed. It has been shown previously that the SABRE enhancement has a linear dependence with para-hydrogen enrichment. Therefore to overcome this, the enhancements were measured at different *para*-hydrogen concentrations, to ensure there would be no radiation dampening, and subsequently these data were scaled to predict the enhancement at 100% *para*-hydrogen. Figure S3 proves that the magnitude of the signal causes the anti-phase character, as by re-shaking a sample without refreshing the p-H_2 the enhancement decreases as the *para* form converts back to the *ortho* form. It can be seen that by the fourth shake the peaks are in-phase albeit with lower overall signal gain.

The purity of the p-H_2 generator used in this research has been previously investigated in detail for a range of different interconversion temperatures.[3] Since p-H_2 is the lowest energy state for H_2 to be in if the gas is cooled and subsequently passed over a paramagnetic catalyst then the *para* state is preferred. The colder the temperature used during conversion, the higher the purity of p-H_2. In the previous study the purity was determined by obtaining NMR spectra of H_2 gas dissolved in 0.6 mL of toluene-d_8 obtained at different interconversion temperatures. These were subsequently fitted with the known equation that dictates the purity.[3] The SABRE enhancement is linked to p-H_2 purity by the linear expression in Equation S2.

$$\varepsilon_S = \frac{4}{3}En_p - \frac{E}{3} \quad \text{(S2)}$$

Where E is an efficiency parameter which can be determined from the gradient of the line when ε_S is plotted against the proportion of p-H_2 (between 0.25 at room temperature and

above up to 1 for temperatures approaching zero). This relationship has been experimentally shown to hold up to 99 % p-H_2 and therefore by taking a few different purities and measuring the SABRE enhancement, it is possible to estimate the enhancement at 99 % p-H_2.[3] Therefore this can be of use here for when the signal strength can cause anti-phase character, the purity of the gas can be reduced and the maximum enhancement estimated easily.

The different interconversion temperatures were achieved through the use of a closed cycle helium compression unit which is capable of cooling down to 7 K coupled with a heater on a feedback loop which allows any temperature. p-H_2 was added to the samples at different purities but always at 4 bar (absolute) pressure, before being shaken for 10 seconds in a PTF of 61 G. After shaking the samples were placed inside the spectrometer and a 90° pulse and acquire sequence was carried out in a single scan. The corresponding thermal reference spectra were taken in a single scan also, which were subsequently used to determine the enhancement values.

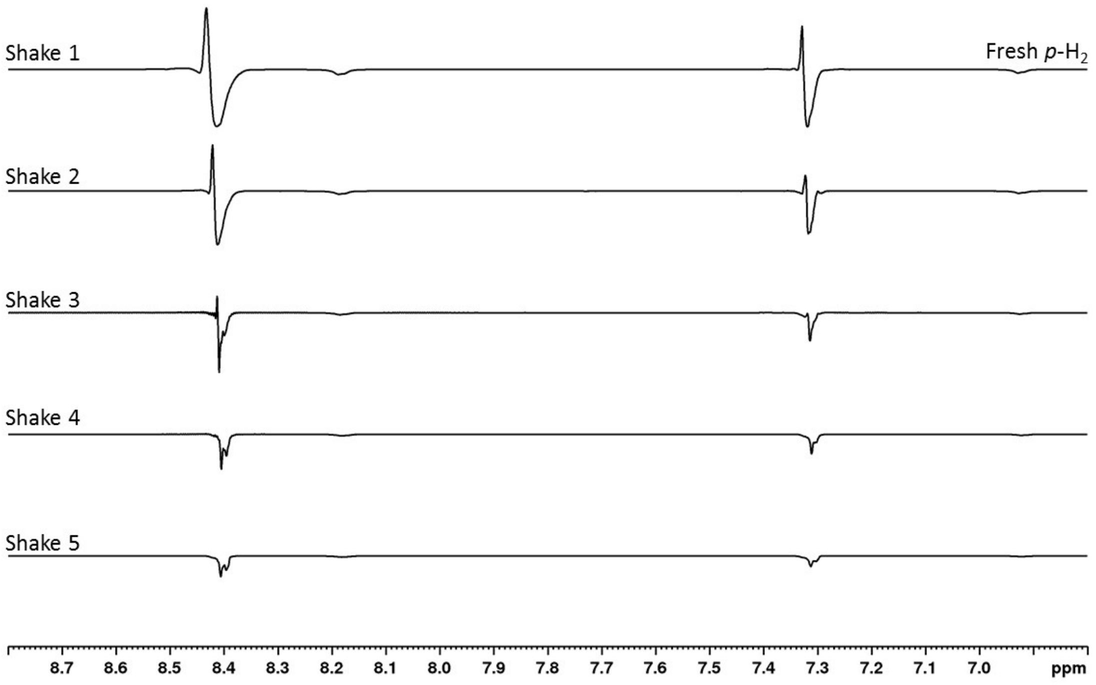

Figure S3 - Radiation dampening during S&Ds with substrate A catalyst 2 with 99.9 % pH_2.

Conversion T (K)	[p-H_2] (%)	Enhancement factor
99	40.1	-175.43 ± 1.40
80	49.7	-311.37 ± 9.75
67	59.9	-446.41 ± 2.51

Table S2 - Enhancements for [Ir(H)$_2$(2)(B)$_3$]Cl with varying concentrations of p-H_2 **as determined previous from the corresponding temperatures.**

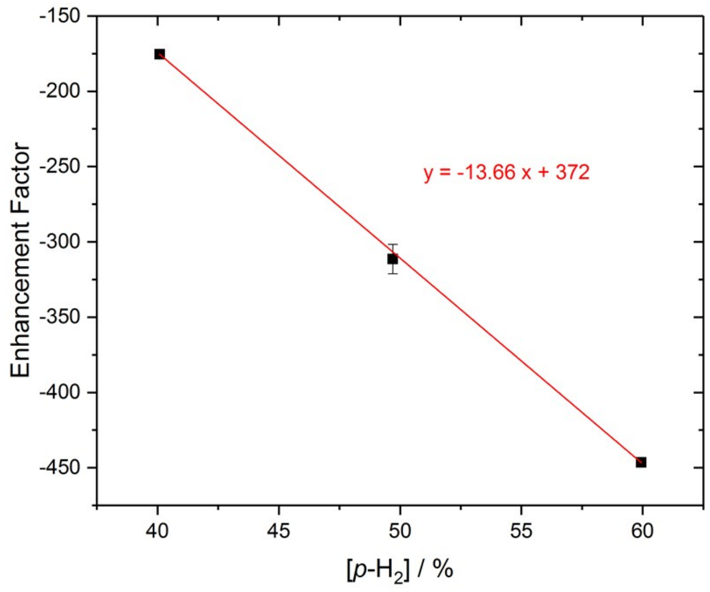

Figure S4 - Plot of enhancement factor against p-H_2 concentration for NHC 2 + substrate B.

This method was also repeated for the fully deuterated analogue of NHC **2** (**d^{34}-2**) and substrate **B**. This experiment was carried out by another experimenter using a manual 61 G shaker, therefore both the non-deuterated and deuterated analogues had to be assessed together.

Conversion T (K)	[pH$_2$] (%)	Enhancement of 2	Enhancement of d^{34}-2
140	29.0	-53.79±1.37	-63.95±1.88
99	40.1	-210.83±14.57	-264.03±10.35
80	49.7	-323.22±14.43	-480.95±20.36
67	59.9	-443.02±28.06	-668.96±31.15
57.5	69.9	-618.91±56.42	-839.32±22.22

Table S3 – Enhancement Factors for substrate B and NHC 2 and d^{34}-2.

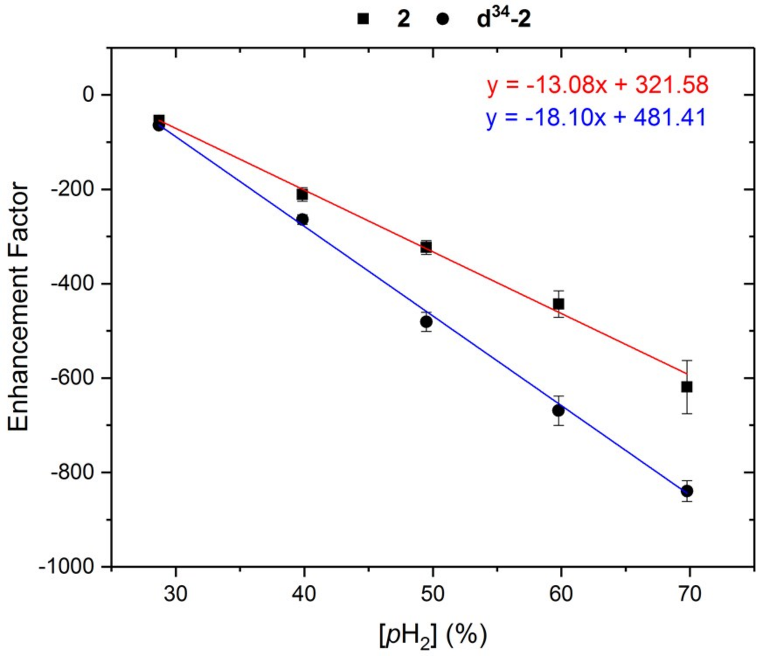

Figure S5 - Plot of enhancement factor against p-H_2 concentration for substrate B and NHC 2 and d^{34}-2 using 61 G shaker

3. Substrate Dissociation

Exchange Pathways

Exchange spectroscopy (EXSY) was used to calculate the rate of dissociation (k_d) of the bound substrate. In this experiment, the *ortho* proton site of the substrate bound *trans* to the hydrides in the active catalyst (A below) was selectively excited. The resulting free induction decay then contains signals from this site and any site that it connects with through exchange. In this case the process of dissociation from the iridium centre results in the detection of an additional signal for the corresponding resonance in the free substrate in solution. In order to encode data that would allow the rate of this process to be measured, a series of measurements were made at set time increments for a given temperature to do this. The result is a fall in intensity of the excited peak and a growth in intensity of the exchange peak with delay time.

The exchange pathway leading resulting in this process in $[Ir(H)_2(SIMes)(sub)_3]Cl$ is shown below. 'X' is used to denote the selectively-excited bound substrate and 'Y' the unlabelled free substrate that binds to the complex as it is replaced. The rate constants k_a and k_d are used to describe the rate of association and the rate of dissociation respectively. However, only the slow step k_d is well defined.

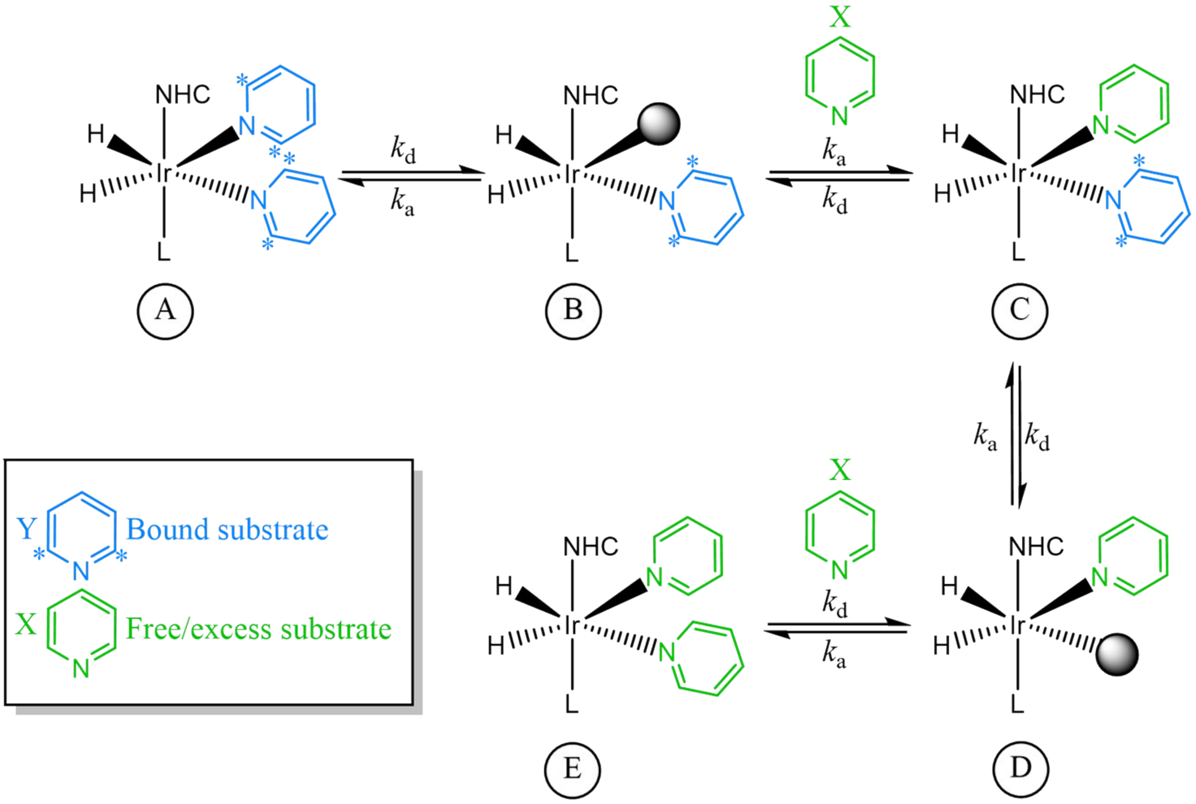

Figure S6 - Exchange species for $[Ir(H)_2(SIMes)(sub)_3]Cl$ where K_a is the rate of association and K_d the rate of dissociation

The concentration of each of these species can be assessed using a differential model in conjunction with a time interval ∂t according to the following formulae. In this case $[A]_t$ defines the concentration of [A] at the time point t ($[A]_t$) while $[A]_{t+\partial t}$ defines it at the following time point t+δt. In this case species A reacts to lose one of the substrate ligands which means the net rate of formation of B is $2k_d[A]_t\partial t$ and hence the concentration of

$[A]_{t+\partial t}$ becomes $[A]_t - 2k_d[A]_t\partial t$, however A also forms from the reaction of intermediate B with X and hence the overall equation for $[A]_{t+\partial t}$ is given by Equation S3.

$$[A]_{t+\partial t} = [A]_t + k_a[B]_t[X]_t\partial t - 2k_d[A]_t\partial t \qquad \text{(S3)}$$

In a similar way, we assess the concentration of the intermediate $[B]_{t+\partial t}$. It forms from A and C with a proportion $2k_d$ and k_d in recognition of the fact they contain two and one molecules of initially excited substrate respectively. Species B is depleted by formation of A and C which gives rise to Equation S4.

$$[B]_{t+\partial t} = [B]_t + 2k_d[A]_t\partial t - k_a[B]_t[X]_t\partial t + k_d[C]_t\partial t - k_a[B]_t[Y]_t\partial t \qquad \text{(S4)}$$

Complex C can then be profiled by Equation S5.

$$[C]_{t+\partial t} = [C]_t + k_a[B]_t[Y]_t\partial t - 2k_d[C]_t\partial t + k_a[D]_t[X]_t\partial t \qquad \text{(S5)}$$

According to Figure S6, C then leads to intermediate D. Its behaviour is identical to B, excepting the fact we need to take into account which groups were initially excited. Therefore complex D can be characterised by Equation S6.

$$[D]_{t+\partial t} = [D]_t + k_d[C]_t\partial t - k_a[D]_t[X]_t\partial t + 2k_d[E]_t\partial t - k_a[D]_t[Y]_t\partial t \qquad \text{(S6)}$$

The final species E is NMR silent in this experiment, and forms from D, giving rise to Equation S7.

$$[E]_{t+\partial t} = [E]_t + k_a[D]_t[Y]_t\partial t - 2k_d[E]_t\partial t \qquad \text{(S7)}$$

The concentration of X and Y are therefore critical in this model and given by Equations S8 and S9.

$$[X]_{t+\partial t} = [X]_t + 2k_d[A]_t\partial t - k_a[B]_t[X]_t\partial t + k_d[C]_t\partial t - k_a[D]_t[X]_t\partial t \qquad \text{(S8)}$$

$$[Y]_{t+\partial t} = [Y]_t + 2k_d[E]_t\partial t - k_a[B]_t[Y]_t\partial t + k_d[C]_t\partial t - k_a[D]_t[Y]_t\partial t \qquad \text{(S9)}$$

A spreadsheet was created in Microsoft Excel using the formulae above to model the values of $[A]_t$ and $[X]_t$ that come from the EXSY data. Starting inputs were the relative concentrations of the complex (A) and free substrate (Y) in solution.

For example, the samples used for these EXSY experiments contain a 50 mM concentration of $[Ir(H)_2(SIMes)(sub)_3]Cl$ (complex A) and a 10-fold excess of the substrate. Three of these substrate molecules are bound into the SABRE-active complex whilst the other 7 remain in the surrounding solution (Y) and therefore, a concentration of 0.035 mmol dm^{-3} is used for Y. The initial values of k_a and k_d were estimated with a time increment δt of 0.001 s.

The simulated data were then fitted to the experimental data using the Excel's Solver package to minimise the sum of the square difference. It was assumed that each value had the same uncertainty and therefore an unweighted least squares method was employed. A plot of the simulated data was compared with the experimental data to ensure a suitable fit.

A jackknife approach was used to quatify the accuracy of these rate constants which lie in the region of 1% standard error.

Data

	k_d (s^{-1})			
Temp (K)	A	B	C	D
250	---	---	---	0.0101
255	0.1089	0.0821	0.0784	0.0246
260	0.2479	0.1872	0.1731	0.0549
265	0.5431	0.4054	0.3960	0.1281
270	1.0415	0.7547	0.8327	0.2917
275	2.0365	1.5145	1.5885	0.6440
280	4.4560	2.6090	2.8510	1.4042
285	8.8752	6.6854	3.9171	2.6575
290	14.6521	12.2862	---	5.3359
295	---	21.4612	---	10.0709
300	---	---	---	21.4745

Table S4 - Calculated values of k_d for substrates A-D for catalyst 1

	k_d (s^{-1})			
Temp (K)	A	B	C	D
255	---	0.0182	0.0179	---
260	0.0319	0.0423	0.0435	0.0171
265	0.0942	0.1036	0.1032	0.0410
270	0.1935	0.2305	0.2290	0.0947
275	0.4569	0.4908	0.4867	0.2437
280	1.1073	1.1169	1.0915	0.5983
285	1.5724	2.0090	1.7576	1.0221
290	3.1734	3.5985	3.3557	1.7756
295	6.0351	6.7503	6.3944	3.5381
300	---	12.6376	---	---

Table S5- Calculated values of k_d for substrates A-D for catalyst 2

	k_d (s^{-1})			
Temp (K)	A	B	C	D
260		0.0116		
265	0.0200	0.0252		
270	0.0496	0.0575	0.0496	0.0163
275	0.1351	0.1272	0.1148	0.0386
280	0.3800	0.2946	0.2764	0.1085
285	0.4720	0.5915	0.5697	0.2377
290	1.0382	1.0139	0.9766	0.4952
295	2.2870	2.0642	1.9345	1.0496
300		3.8771		

Table S6 – Calculated values of k_d for substrates A-D for catalyst 3

4. Temperature Enhancements

Variation of enhancement with temperature were studied using an automated flow system[3,4] equipped with a specially designed flow cell. This flow cell contains a sample chamber surrounded by a water jacket which is connected to a variable temperature water bath capable of reaching temperatures from 4°C – 100°C with a view to extending this by using different coolants. The cell is designed such that the water jacket surrounds the sample entirely (Figure S7).

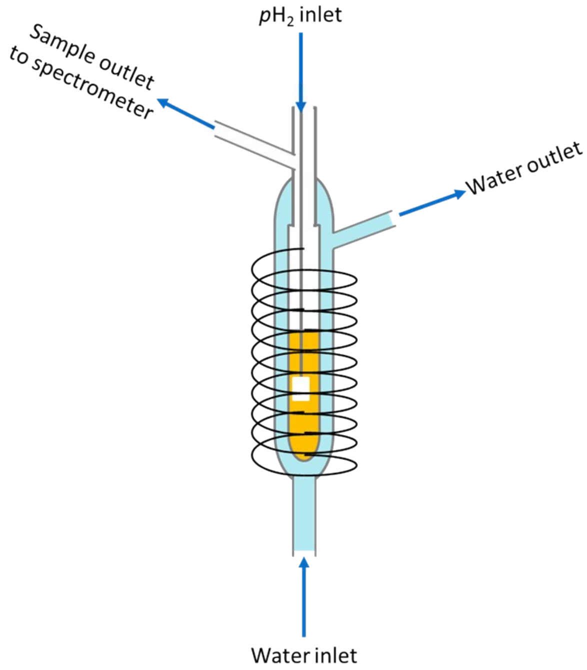

Figure S7 - The flow cell used for the variable temperature study

The complete flow system consists of the sample chamber (pictured in Figure S7) placed inside a solenoid which allows the use of a variable PTF field. p-H_2 is introduced into the sample by bubbling 4 bar (absolute) into the solution via a porous frit. The system is fully automated with the parameters being controlled by the spectrometer software. The transfer of the solution after polarisation was done pneumatically under a pressure of N_2 gas. The sample was extracted by a tube that is placed inside the solution and once pressurised is allowed to travel to the spectrometer. Since the transfer is controlled by the spectrometer software, the spectrum can be immediately acquired upon arrival of the sample, or after a user defined delay, termed the settling time (0.1 s was used here). The SABRE enhancements were measured in the temperature range 280 K - 320 K. For each measurement a bubbling time of 10 seconds was used with a 0.1 s settling delay. In each case the temperature of the spectrometer was matched to the temperature of the mixing chamber. The transfer time used here is on the order to 2 seconds. In between measurements the sample was allowed to rest inside the heated mixing chamber for 5 minutes to ensure equilibrium temperature had been reached. However, we note that this time could be most likely reduced, but was deliberately set to be a significantly long equilibrium time. In all cases a PTF of 65 G was applied to the system during the bubbling stage and subsequently switched off during transfer and acquisition. For all samples at all temperatures 5 hyperpolarised repeat measurements were obtained using a single scan 90°

pulse and acquire, followed by a thermal reference scan where the sample was transferred without bubbling and allowed to reach thermal magnetic equilibrium before measurement.

	Enhancement Factor			
Temp (K)	A	B	C	D
280	-181	-112	-121	-46
285	-172	-128	-127	-59
290	-138	-123	-115	-50
295	-99	-100	-89	-46
300	-39	-67	-54	-37
310	-4.8	-15	-10	-15
320	---	-2.7	-1.1	-3.4

Table S7 - OrthoH Enhancement Factor at different temperatures for the substrates A-D for catalyst 1

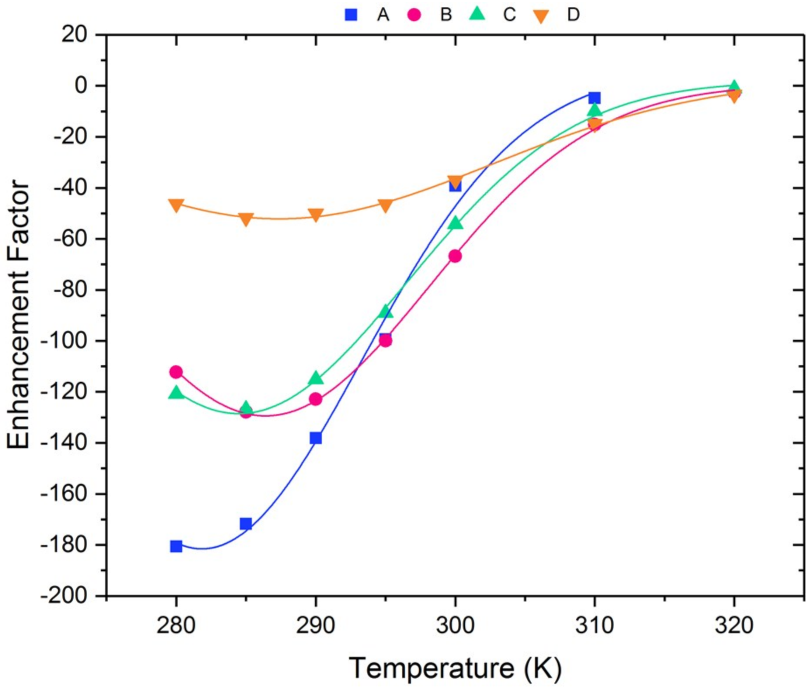

Figure S8 - Variation in enhancement with temperature at OrthoH for the substrates A-D and catalyst 1

5. Calculating Gibbs free energy

The Gibbs free energy (ΔG≠) was calculated using the linear form of the Eyring-Polanyi given by Equation S10.

$$ln\frac{k}{T} = \frac{-\Delta H^{\neq}}{R}.\frac{1}{T} + ln\frac{k_B}{h} + \frac{\Delta S^{\neq}}{R}$$

(S10)

Using this equation, $\frac{1}{T}$ was plotted against $ln\frac{k}{T}$ and the enthalpy and entropy of activation was be determined from the gradient ($-\frac{\Delta H^{\neq}}{R}$) and the intercept ($\ln\left(\frac{k_B}{h}\right) + \frac{\Delta S^{\neq}}{R}$) (Figure S9).

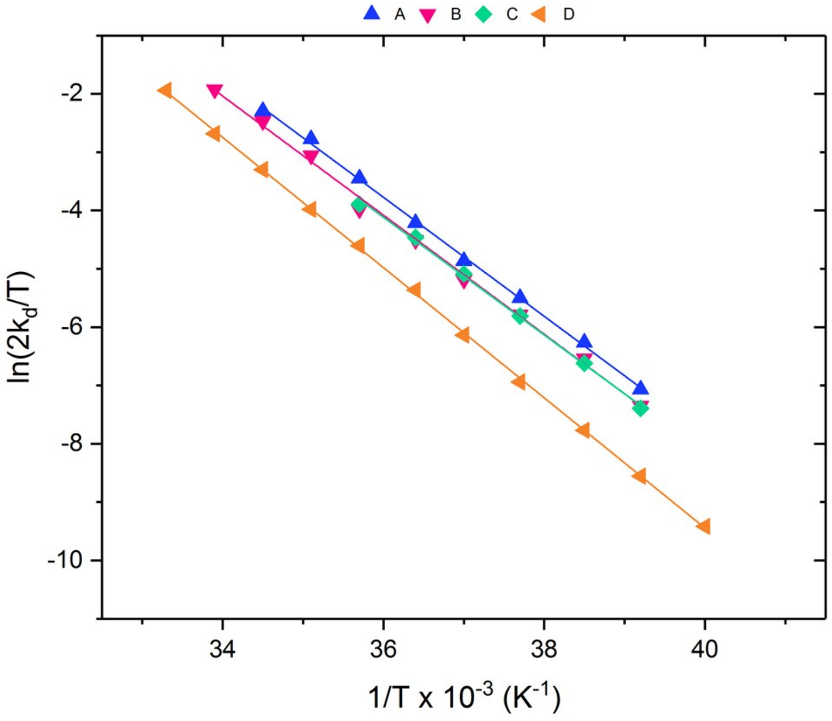

Figure S9 - Eyring-polanyi plot for substrates A-D and catalyst 1

Finally, the free energy of activation, ΔG≠, at 298 K, kJ mol^{-1} from this data was calculated using Equation S11.

$$\Delta G^{\neq} = \Delta H^{\neq} - T\Delta S^{\neq}$$

(S11)

	ΔG≠ (298 K, kJ mol^{-1})		
Substrate	1	2	3
A	62.08	65.64	68.14
B	62.82	65.43	68.46
C	62.94	65.56	68.51
D	64.44	66.86	69.90

Table S8 – Gibbs free energy for each of the substrate and catalyst combinations

6. T_1 relaxation

Calculation

T_1 values were measured using a standard 2-D inversion recovery experiment.

The values quoted are the *ortho* proton of the substrate in the free material. The raw data, fit and value will error are presented below at 298 K unless otherwise stated. Figures S10-S13 provide typical data for catalyst 1

Catalyst 1

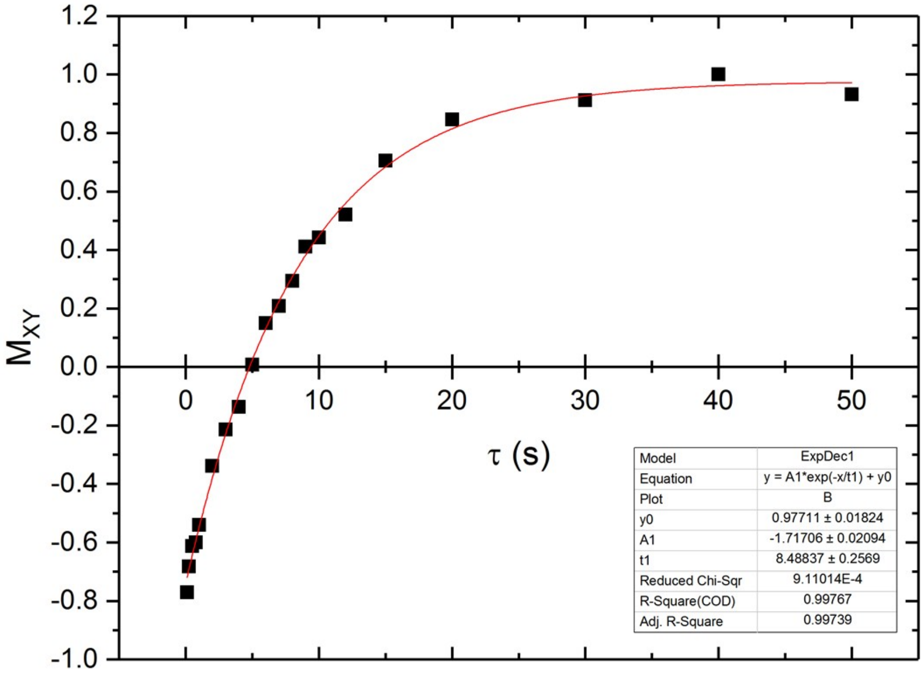

Figure S10 - T_1 for substrate A

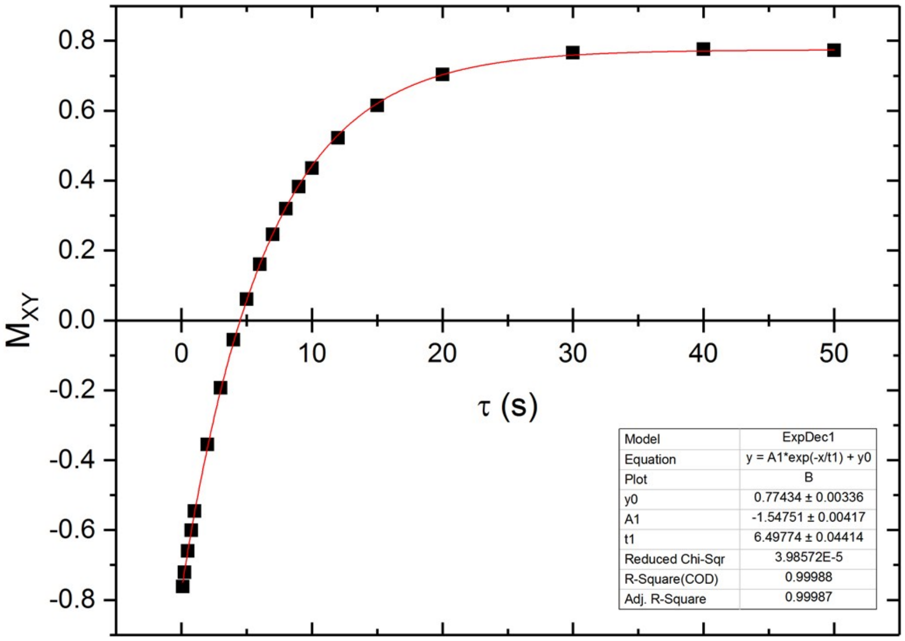

Figure S11 - T_1 for substrate B

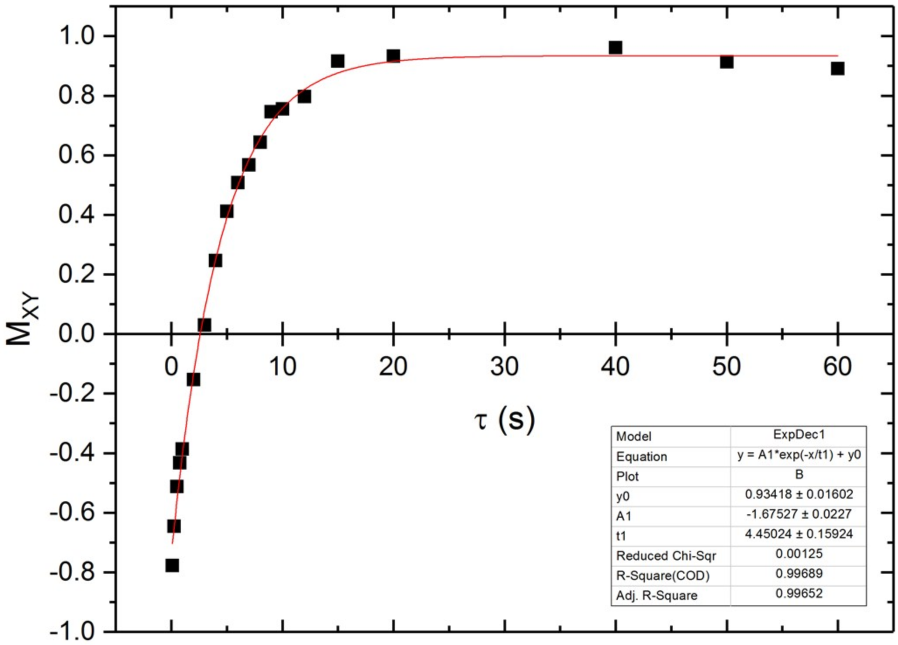

Figure S12 - T_1 for substrate C

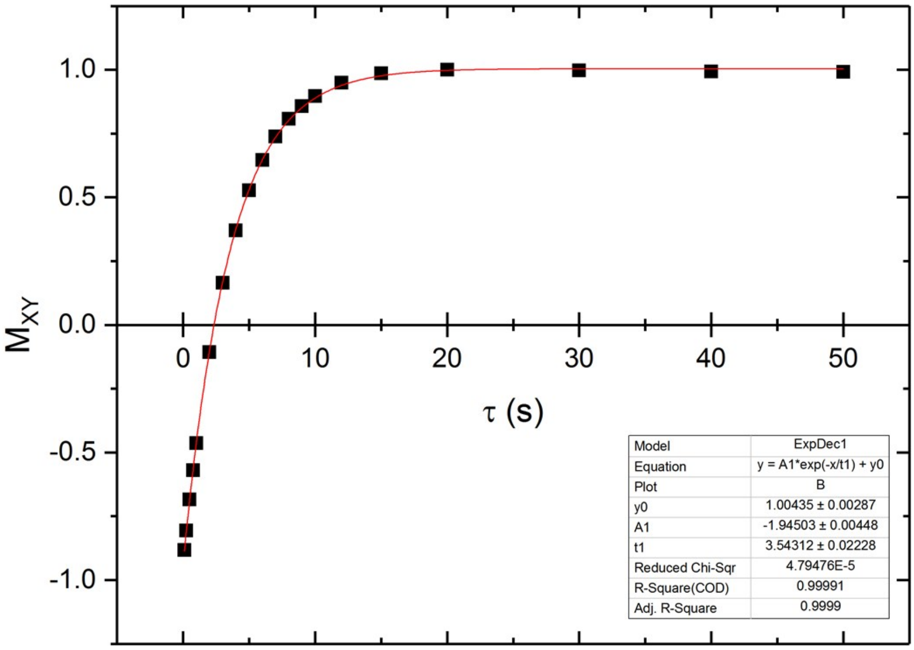

Figure S13 - T_1 for substrate D

Substrate A

The T_1 for substrate **A** was calculated using a Hyperpolarised T_1 pulse program. This is a single-shot experiment where various pulse durations are applied to the sample, yielding different flip angles. The first pulse has a small angle, with subsequent pulses having progressively larger durations. These have been calculated to provide the same signal on each acquisition in the absence of relaxation, meaning the signal decay relates to the decay of the hyperpolarised signal.[5]

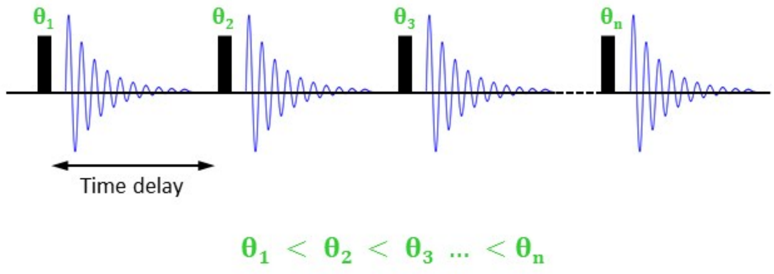

Figure S14 - A single-shot pulse sequence for measuring the lifetime of hyperpolarisation

Time is plotted against the bulk magnetisation of the system, a single exponential was fitted to the data in order to obtain the T_1 value. However, rather than the bulk magnetisation returning to 1 (from -1) as in normal inversion recovery T_1 measurements, the bulk magnetisation for hyperpolarized T_1 values instead returns to zero. In SABRE enhancements, the hyperpolarized signal is shown as negative due to the selective population of the spin states of the proton. Therefore when the hyperpolarised T_1 is measured, the magnetisation becomes less intense as it returns back to thermal equilibrium. A typical plot is shown in Figure S15.

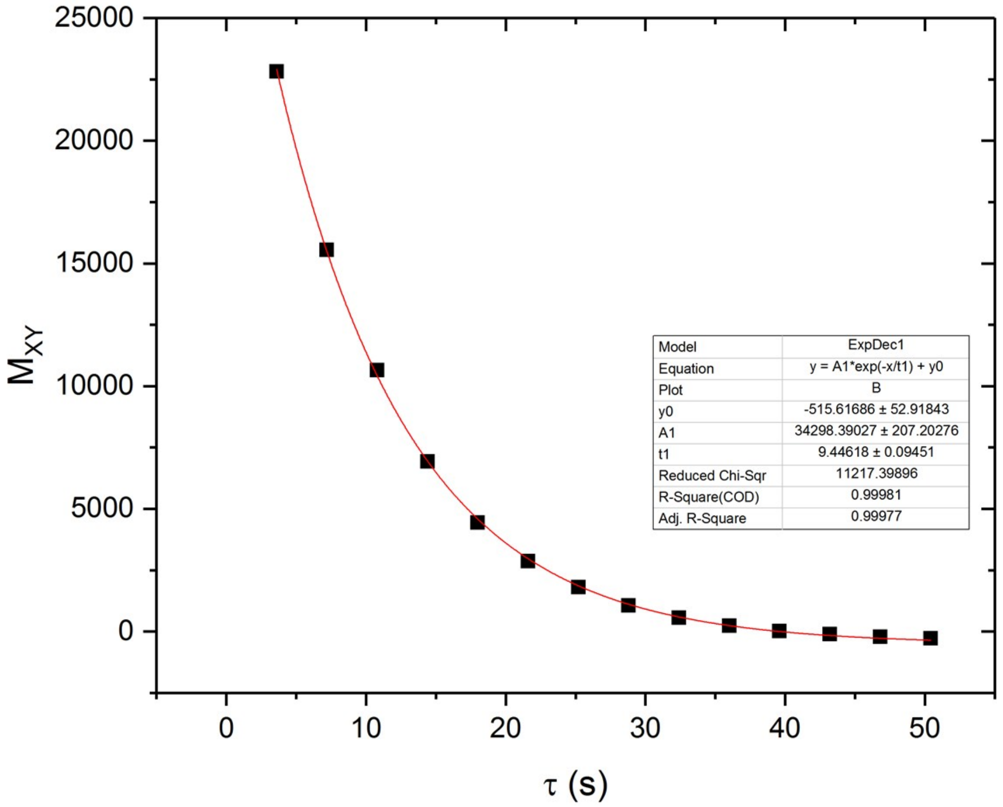

Figure S15 - Hyperpolarised T_1 for substrate A

7. pK_a Measurements Using NMR

There are several methods for determining the pK_a of a substrate in water, in this work we have selected to use the same NMR method in methanol solution as it provides a fast and accurate method to gain an indication of reagent basicity in this solvent.[6] When the substituted pyridines are added to methanol with HCl to control the pH of the solution there are two possible forms that exist in solution shown in scheme 1.

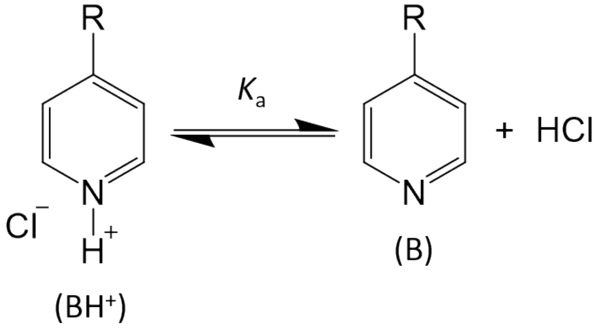

Scheme 1 – The two possible forms of the substituted pyridines in methanol with added hydrochloric acid to control the pH of the solution.

The ionisation constant K_a for the pyridine substituted acids can be described by Equation S12.

$$K_a = \frac{[HCl][B]}{[BH^+]} \quad \text{(S12)}$$

The equation for pK_a can then be written in the form of Equation S13; note throughout we use pK_a even though the work is completed in methanol.

$$pK_a = pH + log\left({[BH^+]}/{[B]}\right) \quad \text{(S13)}$$

This form of the equation can be updated for use in NMR as the concentrations can be exchanged for the fractional populations (x_B and x_{BH^+}) for each form which gives Equation S14.

$$pK_a = pH + log\left({x_{BH^+}}/{x_B}\right) \quad \text{(S14)}$$

In an NMR spectrum the expected chemical shift for the two forms would be different as the presence of the proton in the charged case will typically shift the resonance down field. Since NMR cannot detect chemical change that occurs on a faster timescale than the NMR experiment it would be expected that there would be a single observed peak accounting for both forms. However the location of this peak will be a weighted average which is dependent on the relative populations and the chemical shift of the pure acidic and basic forms. Therefore if the chemical shift in strong acidic conditions (δ_A) and strong basic conditions (δ_B) are known then the measured chemical shift (δ_m) can be determined using equation S15.

$\delta_m = x_{BH^+}\delta_{BH^+} + x_B\delta_B$

(S15)

Since x_{BH^+} and x_B are two fractional populations then they must sum to 1, and thus an equation for the proportion of the deprotonated form (x_B) can be written in the form of equation S16.

$x_B = 1 - x_{BH^+}$

(S16)

By substituting Equation S16 into Equation S15 and subsequently rearranging, a simplified equation for the relative population of the protonated form of the molecule can be determined, as shown in Equation S17.

$$x_{BH^+} = \frac{\delta_m - \delta_B}{\delta_A - \delta_B}$$

(S17)

In an analogous manner an equation for the fractional proportion of the deprotonated form can be expressed as Equation S18.

$$x_B = \frac{\delta_A - \delta_m}{\delta_A - \delta_B}$$

(S18)

Therefore by substituting equations S17 and S18 into equation S14 and rearranging gives an expression for the measured chemical shift as a function of pH, where pK_a, δ_A and δ_B are constants for a given molecule (Equation S19).

$$\delta_m = \frac{\delta_A 10^{(pK_a - pH)} + \delta_B}{1 + 10^{(pK_a - pH)}}$$

(S19)

It is therefore possible to measure the chemical shift of a molecule by NMR for solutions of different pH levels and fit this data using Equation S19 to be able to determine the pK_a of the molecule.

In this study the pK_a values of each of the four target molecules 4-chloropyridine, 4-pyridinecarboxadlehyde, 4-methylpyridine and 4-methoxypyridine have been measured. It is important to note that all of these have been measured in methanol-d_4 in order to mimic the environment they are present in for the hyperpolarisation experiments. Additionally, when the substrate 4-pyridinecarboxaldehyde is introduced to an alcohol (in this case methanol) it will form its hemiacetal and therefore the pK_a of this has been determined.

For each substrate two bulk samples were created each with 10 ml of solvent with around 150 mg of target substrate added with 40 µL of trimethylsilanol introduced as a reference for the chemical shift to ensure there is no drift over time or pH of the sample. To one bulk solution, drops of 1.0 M (and 0.1 M for fine tuning) hydrochloric acid (in methanol-d_4) were added to obtain low pH levels and to the other, drops of potassium hydroxide (in methanol-d_4) were used to reach high pH levels. The pH levels of the solutions were determined using a benchtop pH sensor (Mettler Toledo) which was calibrated before each sample using the two point inbuilt calibration sequence using buffers at pH 4 and pH 7. For each sample a

range of pH values were measured and subsequently extracted and placed into NMR tubes for analysis. The chemical shifts were determined from a simple pulse and acquire using a 90° pulse. For each molecule all of the present ^{1}H resonances were analysed, and the trimethylsilanol peak was recorded, which should be mostly independent of pH. The data measured was fitted with equation 8 using a least squares regression algorithm where the parameters δ_A (chemical shift in acid), δ_B (chemical shift in base) and pK_a were allowed to be free in the fitting routine. These values were determined for each resonance on the target molecule and displayed accordingly in Figures S16-S19 (the raw data of these plots can be found in Tables S9-S12). In all cases the trimethylsilanol signal only varied slightly, changing by a maximum of 0.005 ppm over the complete range of pH values, therefore assuring that there is no drift of the chemical shift.

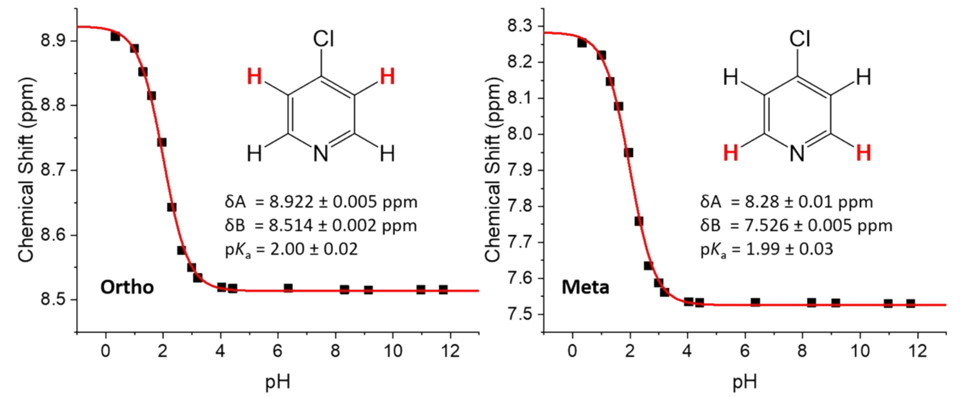

Figure S16 – Observed chemical shift of each peak of the 4-chloropyridine substrate measured as a function of pH.

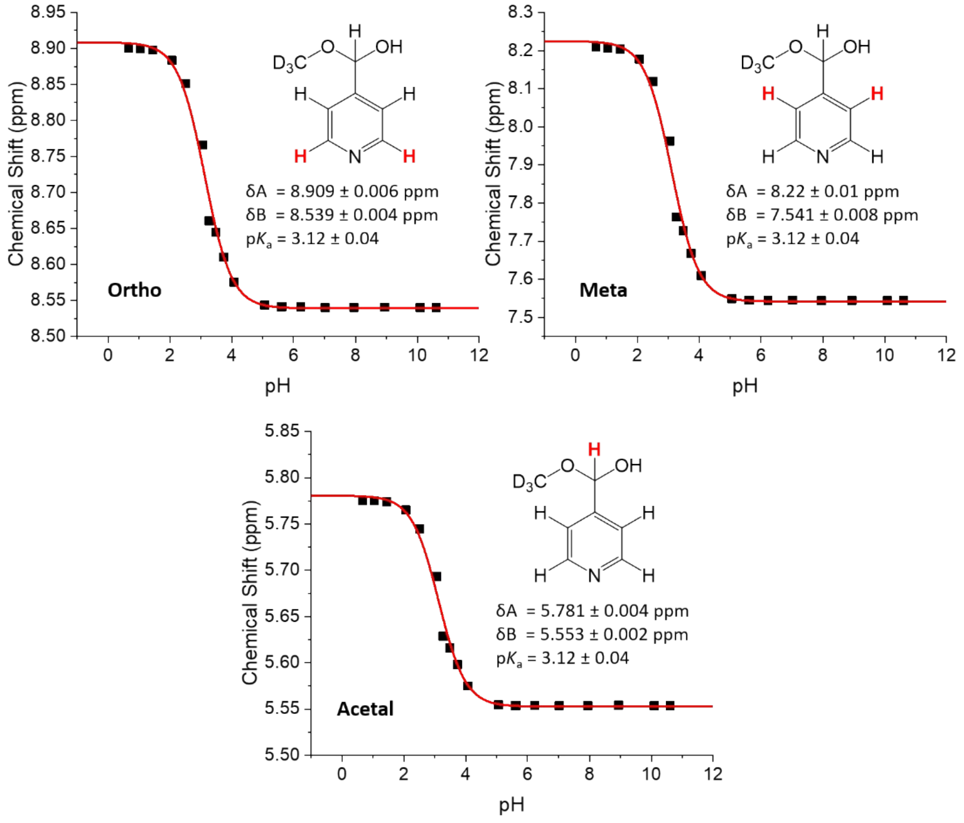

Figure S17 – Observed chemical shift of each peak of the 4-pyridinecarboxaldehyde (hemiacetal form) substrate measured as a function of pH.

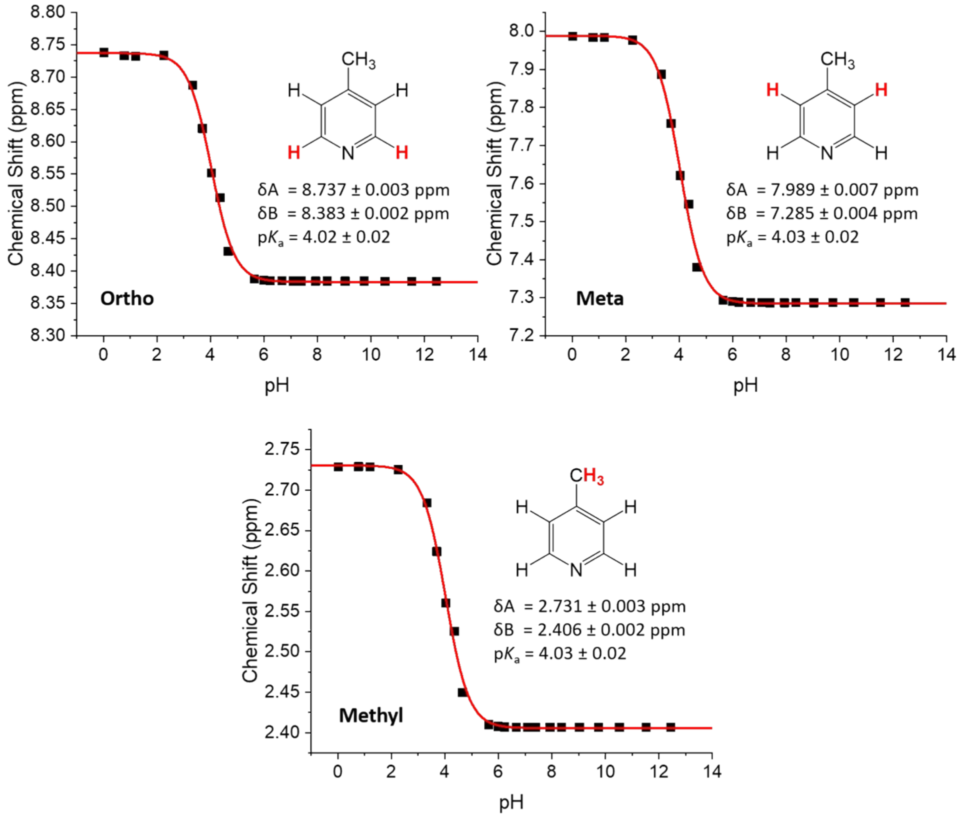

Figure S18 – Observed chemical shift of each peak of the 4-methylpyridine substrate measured as a function of pH.

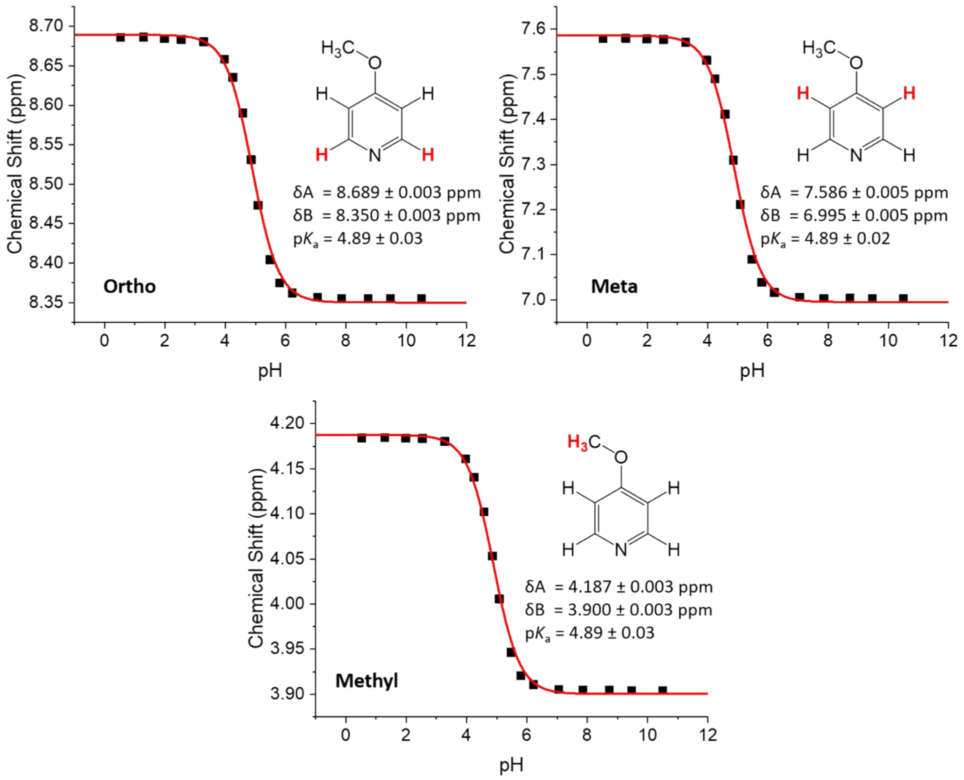

Figure S19 – Observed chemical shift of each peak of the 4-methoxypyridine substrate measured as a function of pH.

pH	Chemical Shift (ppm) trimethylsilanol	Ortho	Meta
0.33	0.02376	8.9069	8.2543
1.00	0.0231	8.8883	8.22003
1.30	0.02282	8.8522	8.14688
1.60	0.022	8.8155	8.0777
1.95	0.02146	8.7431	7.9494
2.30	0.0204	8.6431	7.7591
2.64	0.01973	8.5764	7.63442
2.99	0.01916	8.5501	7.58731
3.20	0.01961	8.5339	7.56095
4.04	0.01884	8.5194	7.53509
4.42	0.0191	8.5176	7.53252
6.35	0.0185	8.518	7.5333
8.31	0.0185	8.516	7.532
9.15	0.0186	8.5155	7.5315
10.97	0.01949	8.5157	7.53
11.75	0.0193	8.5158	7.52987

Table S9 – Observed chemical shifts of each peak of the 4-chloropyridine substrate measured as a function of pH, with trimethylsilanol reference values included.

pH	Chemical Shift (ppm) trimethylsilanol	Ortho	Meta	Acetal
0.67	0.02123	8.9002	8.20902	5.77548
1.05	0.021	8.8995	8.20697	5.77512
1.45	0.02094	8.8979	8.20408	5.77426
2.07	0.02105	8.8835	8.1777	5.7651
2.51	0.02135	8.8512	8.1185	5.74493
3.06	0.02159	8.7661	7.9628	5.69315
3.27	0.0206	8.6605	7.7636	5.62872
3.48	0.02045	8.6448	7.7269	5.61605
3.74	0.02009	8.61	7.6684	5.5981
4.07	0.0223	8.5755	7.6094	5.57501
5.06	0.02242	8.5434	7.5494	5.5547
5.60	0.0225	8.5411	7.54563	5.55364
5.63	0.02244	8.5414	7.54553	5.55359
6.23	0.0223	8.5412	7.54465	5.55369
7.02	0.02237	8.54013	7.5447	5.55355
7.95	0.02235	8.5403	7.54407	5.5536
8.94	0.02207	8.5404	7.54426	5.55413
10.09	0.02242	8.5401	7.54411	5.5536
10.61	0.02242	8.5401	7.54424	5.5535

Table S10 – Observed chemical shifts of each peak of the 4-pyridinecarboxaldehyde (hemiacetal form) substrate measured as a function of pH, with trimethylsilanol reference values included.

pH	Chemical Shift (ppm) trimethylsilanol	Ortho	Meta	CH3
0.02	0.01838	8.73791	7.98736	2.72885
0.77	0.0188	8.73305	7.98467	2.72894
1.20	0.01869	8.73207	7.9841	2.72873
2.26	0.02074	8.7336	7.97742	2.72539
3.33	0.02122	8.6874	7.8877	2.68417
3.70	0.0197	8.6207	7.7583	2.62406
4.03	0.01966	8.5518	7.62087	2.56039
4.36	0.01968	8.5137	7.54574	2.52561
4.66	0.02056	8.43082	7.38023	2.44976
5.64	0.02059	8.388	7.2938	2.40985
5.99	0.02026	8.38597	7.2903	2.40783
6.23	0.02046	8.3853	7.28836	2.40717
6.67	0.02053	8.3851	7.2876	2.40687
7.09	0.02067	8.3848	7.28715	2.40673
7.39	0.02074	8.3848	7.2869	2.40669
7.93	0.02072	8.38456	7.28695	2.40669
8.36	0.02072	8.38481	7.28703	2.4068
9.03	0.02053	8.3845	7.28694	2.40659
9.75	0.02042	8.38457	7.28701	2.4066
10.52	0.02027	8.38426	7.28706	2.40657
11.52	0.02027	8.38432	7.28711	2.40651
12.44	0.019925	8.38428	7.28725	2.40667

Table S11 – Observed chemical shifts of each peak of the 4-methylpyridine substrate measured as a function of pH, with trimethylsilanol reference values included.

pH	Chemical Shift (ppm) trimethylsilanol	Ortho	Meta	OMe
0.53	0.0187	8.6859	7.5795	4.1843
1.29	0.0193	8.6865	7.5798	4.1845
1.99	0.0189	8.6843	7.5785	4.1839
2.54	0.01901	8.6836	7.5775	4.1835
3.28	0.0193	8.6805	7.5712	4.1802
3.97	0.0197	8.6584	7.53113	4.1608
4.25	0.0197	8.6354	7.4901	4.1406
4.58	0.0197	8.59	7.4113	4.1023
4.86	0.0198	8.5312	7.3094	4.053
5.09	0.0195	8.4732	7.21106	4.0056
5.48	0.0198	8.4045	7.0898	3.9464
5.8	0.0196	8.37491	7.0386	3.9205
6.22	0.0204	8.3622	7.01601	3.9106
7.06	0.0204	8.3565	7.0051	3.90537
7.86	0.02044	8.355	7.0031	3.90454
8.73	0.0206	8.355	7.00326	3.9043
9.47	0.0201	8.3549	7.0029	3.9041
10.5	0.02007	8.35495	7.00304	3.90413

Table S12 – Observed chemical shifts of each peak of the 4-methoxypyridine substrate measured as a function of pH, with trimethylsilanol reference values included.

8. References

1. P. J. Rayner, P. Norcott, K. M. Appleby, W. Iali, R. O. John, S. J. Hart, A. C. Whitwood and S. B. Duckett, *Nature Communications*, 2018, **9**.
2. P. M. Richardson, S. Jackson, A. J. Parrott, A. Nordon, S. B. Duckett and M. E. Halse, *Magn. Reson. Chem.*, 2018, **56**, 641-650.
3. P. M. Richardson, R. O. John, A. J. Parrott, P. J. Rayner, W. Iali, A. Nordon, M. E. Halse and S. B. Duckett, *Phys. Chem. Chem. Phys.*, 2018, **20**, 26362-26371.
4. R. E. Mewis, K. D. Atkinson, M. J. Cowley, S. B. Duckett, G. G. R. Green, R. A. Green, L. A. R. Highton, D. Kilgour, L. S. Lloyd, J. A. B. Lohman and D. C. Williamson, *Magnetic Resonance in Chemistry*, 2014, **52**, 358-369.
5. O. Semenova, P. M. Richardson, A. J. Parrott, A. Nordon, M. E. Halse and S. B. Duckett, *Anal. Chem.*, 2019, 10.1021/acs.analchem.1029b00729.
6. C. S. Handloser, M. R. Chakrabarty and M. W. Mosher, *J. Chem. Educ.*, 1973, **50**, 510-511.
